# Conformational Heterogeneity of the HIV Envelope Glycan Shield

**DOI:** 10.1038/s41598-017-04532-9

**Published:** 2017-06-30

**Authors:** Mingjun Yang, Jing Huang, Raphael Simon, Lai-Xi Wang, Alexander D. MacKerell

**Affiliations:** 1 University of Maryland Computer-Aided Drug Design Center, Department of Pharmaceutical Sciences, School of Pharmacy, University of Maryland, Baltimore, Maryland USA; 2 Center for Vaccine Development, Institute for Global Health, School of Medicine, University of Maryland, Baltimore, Maryland USA; 3 Department of Chemistry and Biochemistry, University of Maryland, 8051 Regents Drive, Room, 3500 College Park, Maryland USA

## Abstract

To better understand the conformational properties of the glycan shield covering the surface of the HIV gp120/gp41 envelope (Env) trimer, and how the glycan shield impacts the accessibility of the underlying protein surface, we performed enhanced sampling molecular dynamics (MD) simulations of a model glycosylated HIV Env protein and related systems. Our simulation studies revealed a conformationally heterogeneous glycan shield with a network of glycan-glycan interactions more extensive than those observed to date. We found that partial preorganization of the glycans potentially favors binding by established broadly neutralizing antibodies; omission of several specific glycans could increase the accessibility of other glycans or regions of the protein surface to antibody or CD4 receptor binding; the number of glycans that can potentially interact with known antibodies is larger than that observed in experimental studies; and specific glycan conformations can maximize or minimize interactions with individual antibodies. More broadly, the enhanced sampling MD simulations described here provide a valuable tool to guide the engineering of specific Env glycoforms for HIV vaccine design.

## Introduction

It has been over 30 years since the identification of Human Immunodeficiency Virus (HIV) as the causative agent of Acquire Immunodeficiency Syndrome (AIDS), and despite marked progress in the development of effective chemotherapeutics, the search for a protective vaccine remains elusive. Recent efforts towards identification of vaccine candidates have focused in part on the structural biology of the surface molecules of the HIV virion to understand in greater depth the molecular determinants of key epitopes that could serve as targets for protective antibodies^1–3^. Significant effort has been placed on elucidation of the three-dimensional (3D) structure of the full HIV Envelope (Env) protein that mediates binding and internalization of the HIV virion within susceptible target cells, including macrophages and CD4+ T-cells. In addition to studies that have characterized the structure of the Env protein, recent work has also provided crucial structural information regarding the glycan shield that surrounds the Env protein, and represents a barrier to the accessibility of Env-specific antibodies^4–20^. Given the supporting evidence of Env protein and glycan epitopes as targets of broadly-neutralizing antibodies (bnAb), better understanding of the conformational properties of Env glycans could inform vaccine design^10, 14^.

To date, there have been over 300 crystallographic structures of the HIV gp120 protein deposited in the Protein Databank^21^, many of which include the full Env sequence^4–20^. While these 3D structural studies of the Env protein have provided important insights into putative epitopes of broadly neutralizing antibodies (bNAbs), they provide little information on the potential dynamic nature of the glycan shield conformations, which could impact induction and antigen recognition of bNAbs. Atomic level molecular dynamics (MD) simulations offer great potential in this area. Indeed, a recent X-ray crystallographic study on the glycan shield was supplemented by MD simulations of the glycosylated Env performed at room temperature to obtain additional information on the spatial distribution of the oligosaccharides comprising the glycan shield^6^. While powerful, conformational sampling by MD simulations alone is challenging, a problem that may be overcome by the coupling of MD simulations with enhanced sampling technologies such as Hamiltonian replica exchange (HREX)^22–32^. Recently, novel HREX methods along with improved empirical force fields for polysaccharides have been developed that increase the accuracy of the information from computational methods with respect to the full range of conformations accessible to glycans^33–40^. In addition, the use of glycosidic (GL) cluster analysis allows for uniquely defining the conformations sampled by the glycans with the analysis of 3D volumes sampled by the glycans and antibodies and overlap coefficients (OC) of those volumes^41, 42^, offering insights into the common regions of space sampled by the different moieties.

With the goal of obtaining a better understanding of the conformational properties of the glycan shield of the Env, and the accessibility of the underlying proteins, we undertook a HREX-MD^33, 34^ study of a glycosylated Env^15^ in which the three gp120/gp41 monomers were differentially glycosylated with high mannose (M5 and M9) glycans (Table S1, supporting information). From this simulation, we obtained a model of substantial glycan conformational heterogeneity, wherein we document the presence of an extensive network of interactions between glycans, with implications for the role of the glycan shield with respect to accessibility of the protein surface to the CD4 receptor, CCR5 and CXCR4 co-receptors, and neutralizing antibodies. Analysis of glycan holes, a phenomenon referring to the omission of specific Env glycans^43^, provided further insights towards the role for specific glycans in mediating accessibility to adjacent glycans and the underlying protein. In addition, the range of potential interactions between the Env glycans with the CD4 receptor and anti-Env antibodies was determined. These results highlight the potential of MD methods combined with enhanced sampling for probing the structure and dynamics of HIV Env glycoforms in the context of a methodology where the composition of the system can be rigorously defined and studied in molecular detail at biologically relevant temperatures, with the results used in a predictive fashion to facilitate vaccine design.

## Results and Discussion

To investigate the conformational heterogeneity of the glycans on the HIV Env, simulation models were generated in which one monomer of the Env trimer (M1) had full glycosylation (23 glycans) and the other two monomers being partially glycosylated with 12 (M2) and 8 (M3) glycans, respectively (Table S1 of the supporting information). Asn residues were glycosylated with high mannose M5 or M9 glycans (Figure S1 of the supporting information), with residues for glycosylation selected based on those known to interact with antibodies, as described in the supporting information. The model was generated prior to the recent publication of the low-temperature crystal structure of a glycosylated ENV in which a number of glycans were identified and their conformations characterized^6^. In that study standard MD simulations were also performed^6^. In the present study we expand on the previous work by performing enhanced sampling Hamiltonian Replica Exchange with solute tempering and biasing potential (HREST-BP) simulations^34^. To verify the utility of the enhanced sampling approach we also performed a 400 ns standard MD simulation on the ENV system. As detailed in section S.6 of the supporting information the use of the HREST-BP approach leads to a wider range of conformations being sampled versus standard MD for the ENV glycans. Additionally, HREST-BP simulations of the M5 and M9 glycans linked to either an Asn dipeptide, the gp120 V1V2 loop, or gp120 core, respectively, were performed to investigate the impact of environment on the glycan conformational heterogeneity. Additional analysis focused on the range of sampling by the glycans, including potential inter-glycan and glycan-antibody interactions, as well as the potential for preorganization of the glycans that may impact binding by antibodies. Future studies will focus on the atomic details of the interactions of the glycans with the underlying gp120 and gp41 proteins.

### Relationship of glycan conformational sampling to that observed in experimental structures

Analysis of glycan conformation was initially performed based on the use of glycosidic linkage (GL) clusters, as previously presented^42^ and described in section S.2 of the supporting information. GL clusters are defined based on local minima of the free energy landscape sampled in a specific conformation associated with the glycosidic linkages along with Asn side-chain χ_1_/χ_2_ dihedrals and φ_s_/ψ_s_ dihedrals linking Asn to the glycan. As GL clusters are defined based on dihedral local minima, local conformational changes within a GL cluster can readily occur due to the absence of energy barriers, while larger energy barriers are present between GL clusters.

GL cluster analysis was undertaken on selected glycans (M1-g88, g137, g156, g160, g276, g301, g332 and g392) to determine if conformations sampled in the simulations correspond to those determined in experimental studies, in particular those that are established epitopes of anti-Env antibodies. Tables S5a–h of the supporting information includes the GL clusters for each glycan sampled at a probability of 0.01 or more along with GL clusters observed in a number of experimental structures. In general, the Env trimer system sampled conformations similar to those in the experimental structures. For example, in M1-g88, g160 and g332 the χ1/χ2 dihedrals (the 2^nd^ GL integer) were in GL minima 1 and 2 in the simulation, consistent with that in the experimental structures (Table S5a,d and g) while in M1-g137, g156, g276, g301 and g392, minima 4 and 5 were sampled (Table S5b,c,e,f and h). While for these glycans the two χ1/χ2 minima being sampled correspond to one of the minima used to initiate the simulations, with some glycans (M1-g137 and g406 (result for g406 is not shown)) all four prominent χ1/χ2 minima (Figures S2a,0,1,4 and 5) were sampled in the HREST-BP simulation indicating that the sampling approach is able to cross larger barriers and that the range of GL clusters sampled is being dictated by the local environment of the individual glycans. The ability of the HREST-BP simulation method to cross barriers was supported by potentials of mean force (PMF) for the linkages in the M9-Asn dipeptide glycan showing multiple minima to be sampled for each linkage. Importantly, the highly sampled regions from the HREST-BP simulation are consistent with linkage dihedral values observed in HIV glycan-gp120 crystal structures (see supporting information, Figure S6). With the glycosidic linkages further from the Asn residue, larger variability in the GL cluster minima being sampled occurs with the highest sampled linkers (Prob.> = 0.01) (Table S5). In addition, substantially more conformational diversity was present in the large number of conformations sampled at lower probabilities (i.e., <0.01) consistent with the large total number of GL clusters sampled for each glycan (see Figure S4 of the supporting information).

Concerning GL conformations that have been experimentally observed as targets of antibodies, the assessed glycans sample those conformations to varying degrees in the present simulation (Table S5). Experimental data show that antibody 35O22 bound g88 in GL cluster 11223NN63N6N (Table S5a). In the Env simulation, 11223NN63N6N is sampled 34% of the simulation time, indicating the glycan to be partially preorganized in this preferred conformation in the Env alone. Such preorganization would be expected to facilitate antibody binding^44^, although a large number of other conformations of the glycan are sampled as well. For other glycan conformations observed in published crystal structures (e.g. 112238N85N7N, 11223NN45N6N, 11225NN61NNN and 10223NN83N6N), very similar conformations are sampled in the simulation, with the first four or five linkages proximal to the protein (eg. 11223 and 10223) often observed in one or more GL Clusters (Table S5a). In the case of PGT135, which binds to g392 onto which a M9 glycan was modeled in this study, the results are similar (Table S5h). The antibody binds GL cluster 142234473N65 in the 4JM2 crystal structure^16^. In the Env simulation, sampling of the first 5 linkages in the 14223 conformation is common, though sampling of 44 at positions 6 and 7 is less frequent. However, sampling of 73 at positions 8 and 9 and 65 at positions 11 and 12 is quite common suggesting a more important role of the associated mannose monosaccharides for antibody binding. The 18^th^ GL cluster (142234473565) gives the identical conformation with 1.0% population. Similar trends are observed with other studied antibodies (Table S5). In general, the present results indicate that despite the extensive conformational heterogeneity of the glycans, established anti-Env glycan dependent antibodies largely bind to dominant glycan conformations that are significantly sampled on the Env alone. This is most notable for portions of the oligosaccharide that are tethered and proximal to the Env protein surface (i.e., for the first 4 or 5 linkages in the GL Cluster specification) where the glycans sample conformations with a high probability to which antibodies bind. Previous studies show that glycans and proteins can mutually affect each other’s favorable conformations^45–47^ and thus the glycans may sample conformations that are not typically observed in solution due to their stabilization by interactions with the protein or other glycans^48, 49^. Greater conformational variability is observed in the glycosidic linkages distal to the protein surface, indicating a lower degree of glycan preorganization. This conformational variability is partly arising from the contribution of the flexible 1→6 linkages as revealed in other studies^28, 33, 48, 50–52^. However, the substantial number of conformations in these regions of the glycans observed in the present study indicates that the barriers to conformational transition between them are relatively small and, hence, likely facilitating rapid assumption of the bound conformation upon recognition by antibodies.

The importance of the preorganized states of the N-glycans observed in the antibody-unbound Env can be explained in the context of a mechanism that, in part, includes conformational selection. This mechanism has been extensively studied in the context of many other molecular recognition processes, including binding events involving protein-ligand, protein-protein, protein-DNA/RNA, protein-saccharide, and RNA/DNA-ligand complex formation^41, 53, 54^. In the case of the Env glycans, an antibody would initially interact with the distal regions of the antibodies, which are the most conformationally heterogeneous. However, given the low energy barrier between the different conformations, the glycans may readily assume a local conformation compatible with formation of an initial glycan-antibody complex. Pre-organization of the region of the glycan proximal to the protein surface is then hypothesized to be important to allow for the full Env-antibody interaction to occur without a significant conformational rearrangement of the glycan being required, as a large conformational rearrangement would likely require full dissociation of the antibody. We emphasize that this is the only study to observe the pre-organization phenomenon in saccharide epitopes covalently connected to a protein matrix associated with glycan-dependent antibody binding. Sufficient sampling of glycan conformational space is required to observe the glycan pre-organization phenomenon. The use of enhanced sampling in the context of the HREST-BP method specifically developed to maximize the sampling of oligosaccharides by concurrently using the locally potential biasing and globally temperature scaling algorithms allowed this to be achieved^33, 34, 41, 42^.

### Glycan conformational sampling in Cartesian space

**Figure 1:**
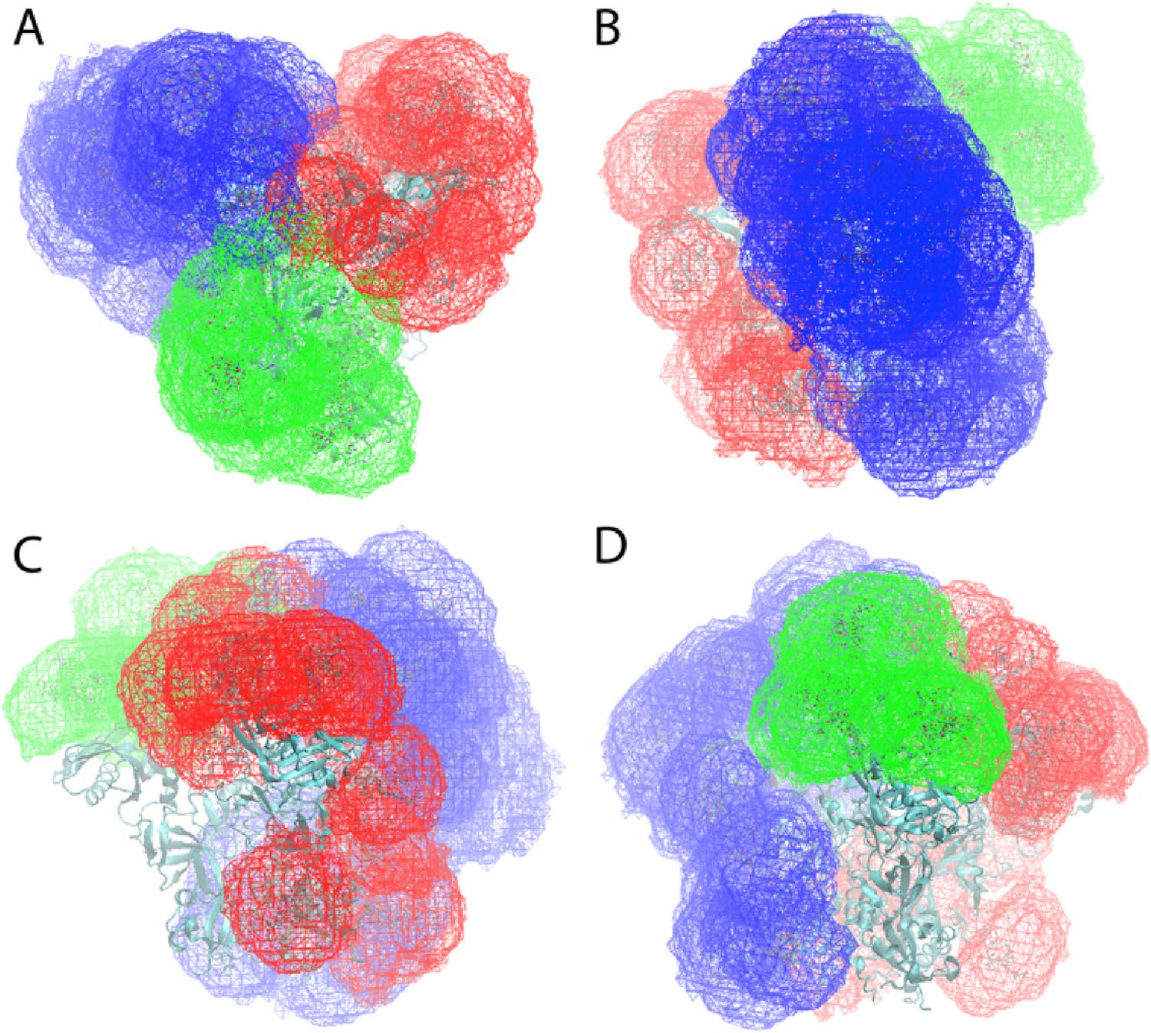
Cartesian volume representations of the range of sampling of all the glycans included in the ENV system. Glycans associated with M-1 are blue, with M-2 are red and with M-3 are green. Shown are (**A**) top view and side views towards (**B**) M-1, (**C**) M-2 and (**D**) M-3. Contour levels are 10^−6^ with the sum of the 1 Å^3^ voxels normalized to 1. The protein is shown in cyan new cartoon representation generated with the program VMD^71^.

Conformational sampling of the glycans was additionally analyzed based on the range of Cartesian space sampled as described in section S.4 of the supporting information. Shown in Fig. 1 are the 3D volumes occupied by all the glycans included in the Env system, with the glycans associated with monomers 1, 2 and 3 colored blue, red and green, respectively. As is evident with M1, when the full range of accessible conformations of the glycans are considered the protein is largely occluded, though some regions of the protein surface are accessible to antibodies throughout the simulation (see below). With the lower extent of glycosylation in M2 and M3, substantial portions of the protein are always accessible, especially considering the accessibility from the side of the Env trimer, as can be seen in Fig. 1C and D.

**Figure 2:**
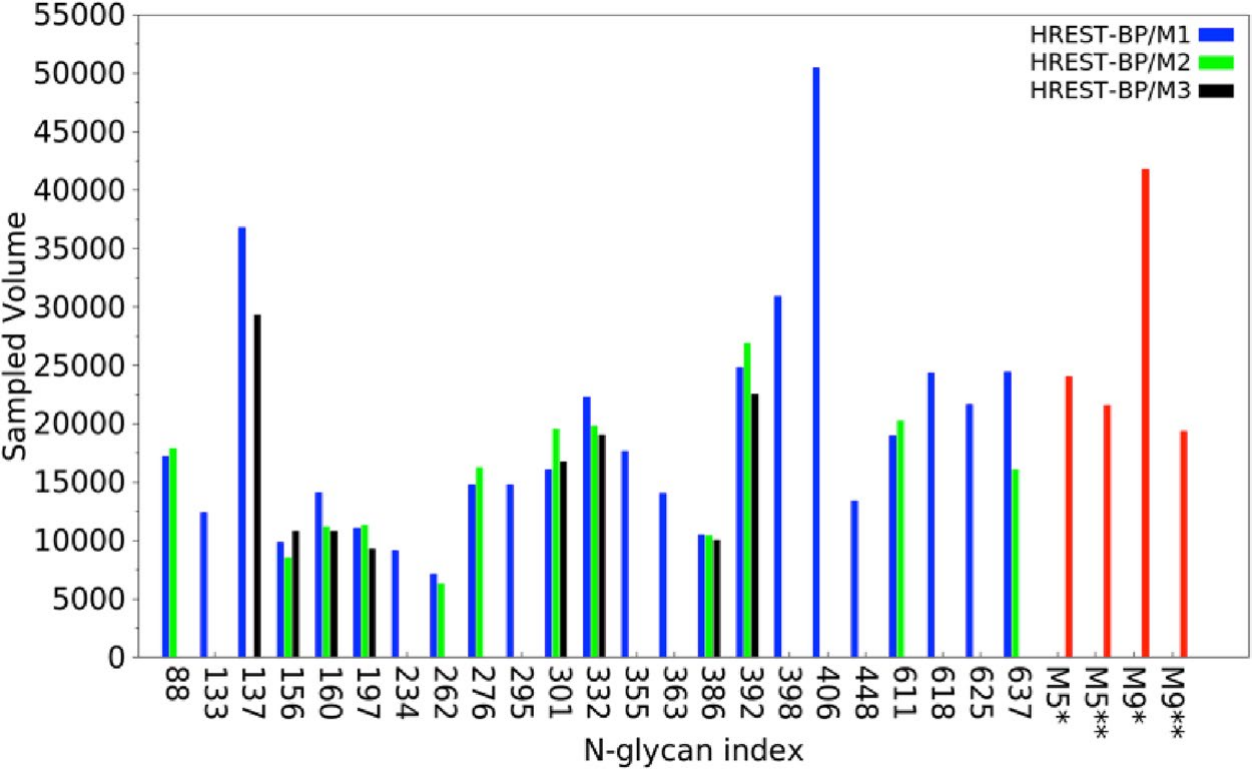
Sampled volume (in Å^3^) of each glycan in the HREST-BP simulations of the Env trimer (HREST-BP/M1, HREST-BP/M2 and HREST-BP/M3), M5 connected to Asn dipeptide (M5*) and the gp120 V1V2 loop (M5**), M9 connected to Asn dipeptide (M9*) and the gp120 core (M9**) models, respectively.

The extent of Cartesian sampling of the individual glycans was evaluated based on the total volume sampled by each glycan (Fig. 2). Notably, the range of volumes sampled varies to a large extent. M9 glycans g332 and g392 tend to sample larger volumes than the M5 glycans, as expected, though some M5 glycans sample substantially larger conformational volumes. The most notable cases are g137 and g406 that, as discussed above, sample 4 GL minima in the χ1/χ2 linkage, which leads to significantly larger sampling of Cartesian space. Interestingly, in the crystallographic study of the glycosylated Env^6^ these glycans were not observed, with the exception of the M5 form in two crystal structures (PDBID: 4NCO^17^ and 5FYL^6^). Alternatively, glycans sampling low volumes in the simulation such as g156, g160, g262, g276 and g386, are observed in all three of the crystal structures reported by Stewart-Jones *et al*.^6^. This consistency may be considered a further validation of the simulation results and emphasizes the utility of the computational approach for investigating the conformational properties of all aspects of Env at physiologically relevant temperatures.

### Glycan-Glycan Interactions

To investigate the extent of interactions between the glycans in the Env system, two types of analyses were undertaken. To determine which glycans have the potential to interact with each other, the overlap coefficient (OC) values of the 3D volume distributions were determined (Equation S2 in the supporting information). In addition, the minimum distance probability distributions of all glycans that come within 5 Å or less based on all non-hydrogen atoms of each glycan were analyzed to identify those glycans that have potential to interact during the simulation as well as the range of minimum distances between the glycans that occurs during the simulation. The distance of 5 Å was selected as it would take into account interactions mediated by a single water molecule.

Results from the OC analysis of the 3D sampled volumes is listed in Table S6 for all pairs with OC > 0, with the number of glycans with the potential to interact with each glycan in monomer 1 listed in Table 1. We found that the number of potential interacting glycan pairs is large, with only M2-88 not interacting with other glycans. For monomer 1 the number of interacting glycans varies from 11 to 2. Of note, glycans with a larger number of interacting partners are generally those known to be essential epitopes for broadly neutralizing antibodies (bNAbs). For example, M1-g137 forms the established epitope for PGT122, and M1-332 is an essential epitope for PGT122 and PGT135 (see below and Table S7 of the supporting information for the antibodies modeled in this study)^6, 15–17^. In general, glycans acting as essential epitopes of antibodies directly interacted with 6 or more adjacent glycans, with the exception being M1-g276, which interacts with Mab 8ANC195^14^, for which only 5 interacting glycans are present. However, M1-g234 is also an essential epitope for that antibody, which may compensate for the lower number of interacting glycans with g276. These results are consistent with previous suggestions^6^ that crowding of glycans would potentially allow for additional glycan-antibody interactions, that may lead to an increased probability of generating bNAbs.

**Table 1:** Number of glycans interacting with each glycan in monomer 1 based on overlap coefficients >0 obtained from the 3D Cartesian volumes.

Glycan	# of interacting glycans
M1-g137	11
M1-g332	11
M1-g406	11
M1-g398	10
M1-g355	9
M1-g133	8
M1-g295	8
M1-g386	8
M1-g392	8
M1-g363	7
M1-g88	6
M1-g156	6
M1-g160	6
M1-g234	6
M1-g301	6
M1-g625	6
M1-g197	5
M1-g262	5
M1-g276	5
M1-g637	5
M1-g618	4
M1-g611	2

Results are sorted from the largest to smallest number of interacting glycans.

**Figure 3:**
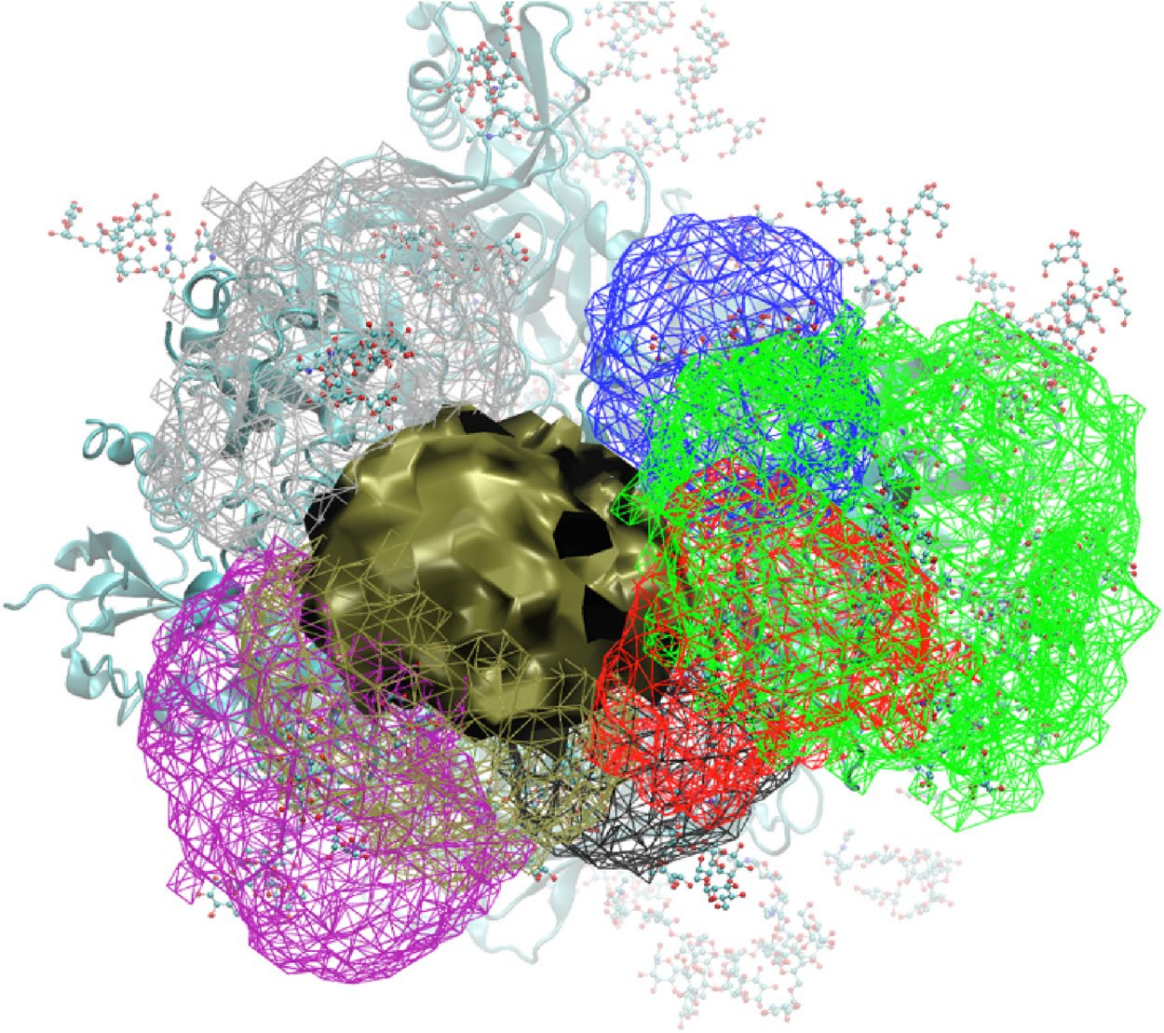
Cartesian volume representations of the range of sampling of all the glycans that interact with M1-g88 (tan solid surface). The volumes for the glycans interacting with M1-g88 are shown in wire representation using the color scheme as follows: M1-g234 (black), M1-g355 (red), M1-g406 (green), M1-g448 (blue), M1-g618 (purple), M1-g625 (tan) and M2-g611 (silver). Contour levels are 10^−6^ with the sum of the 1 Å^3^ voxels normalized to 1. The protein is shown in cyan new cartoon representation and all glycans are in atom-colored CPK representation.

The full range of 3D Cartesian space being sampled by glycans interacting with the 3D volume sampled by M1-g88 is shown in Fig. 3. From the extent of overlap as well as the volumes sampled by the individual glycans, it is clear that the definition of glycan interactions needs to take the full extent of conformational heterogeneity into account. Such heterogeneity will impact the mode of interaction between glycans, as well as the underlying protein. These extensive interactions are associated with the large number of GL clusters (Figure S4 of the supporting information) and 3D volumes (Fig. 2) being sampled by each glycan. Such conformational heterogeneity emphasizes how the flexibility of the glycans in the shield can effectively occlude the majority of the protein from the surrounding environment, and that while the essential epitope of a single antibody may be dominated by a single glycan and conformation, it is evident that a great number of other glycans will influence interactions with that antibody, as presented below.

**Figure 4:**
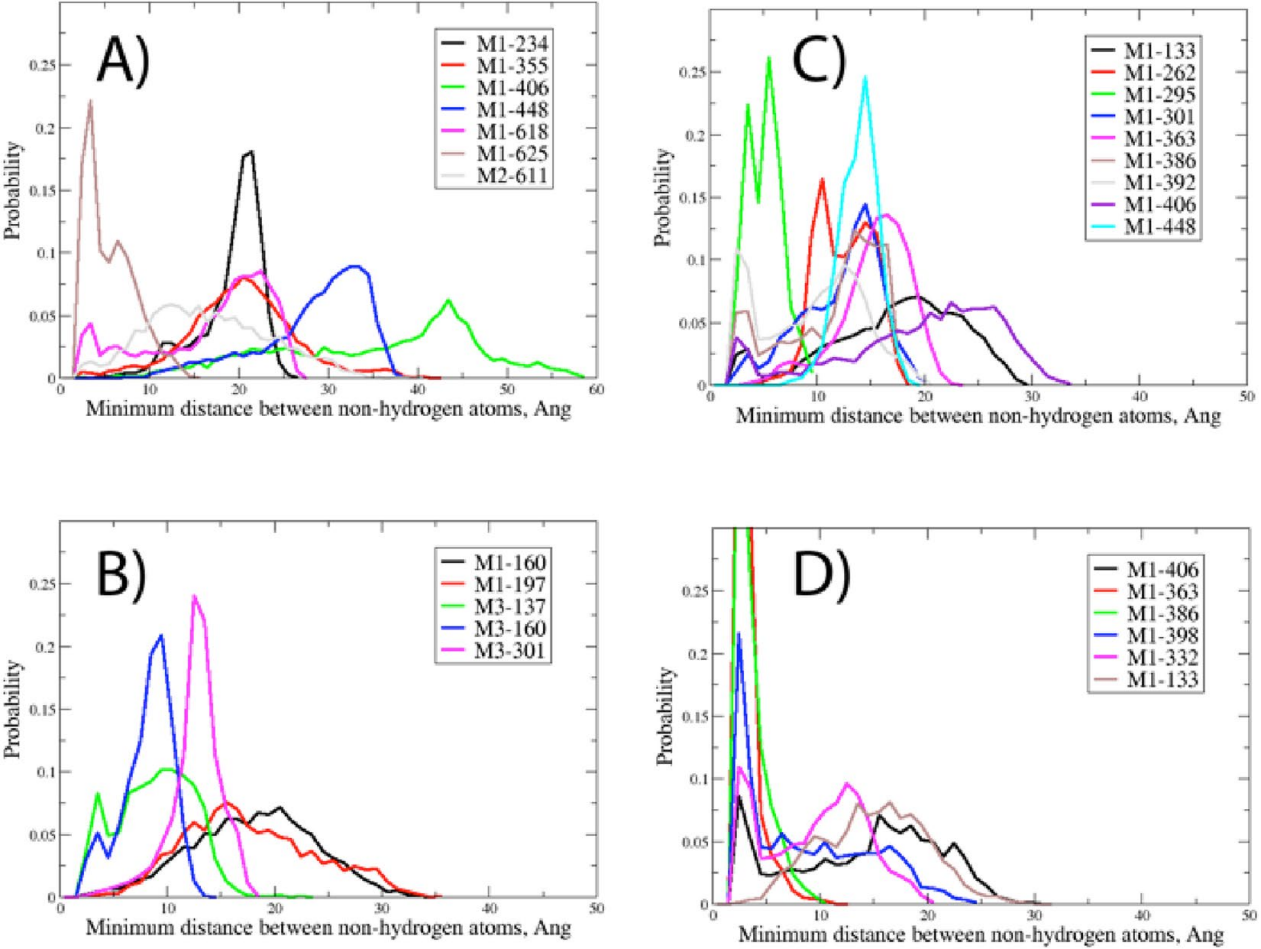
Probability distribution of the minimum distance of all glycans that come within 5 Å or less with the selected glycan for (**A**) M1-g88, (**B**) M3-g156, (**C**) M1-g332 and (**D**) M1-g392.

Analysis of the minimum-distance distribution of glycans that come within 5 Å or less of each other during the simulation are presented for M5 glycans M1-g88 and M3-g156 and M9 glycans M1-g332 and M1-g392 (Fig. 4). Readily evident from the plots are the extreme ranges of minimum interaction distances that occur for many of the glycans. For example, M1-g88 has minimum distances of less than 3 Å to almost 60 Å with glycan M1-g406, indicating that the wide range of conformational space sampled by the individual glycans can lead to large variation in inter-glycan interactions. Even in cases where the glycan pairs are proximal, such as for M1-g88:M1-g625, M1-g332:M1-g295, and M3-g156:M3-g137, each pair of glycans separate by distances of 10 Å or more during the simulation. However, it is evident that in specific cases glycans will maintain close contact, with the best example being between M1-g392 with M1-g363 or M1-g386. These results indicate that the number of specific glycan-glycan interactions is quite extensive, and is much larger than was previously reported for the glycan crystallographic structures, though common interactions are observed in the present and the experimental studies (e.g., M1-g392 with M1-g363 or M1-g386)^6^.

### Protein surface accessibility

**Figure 5:**
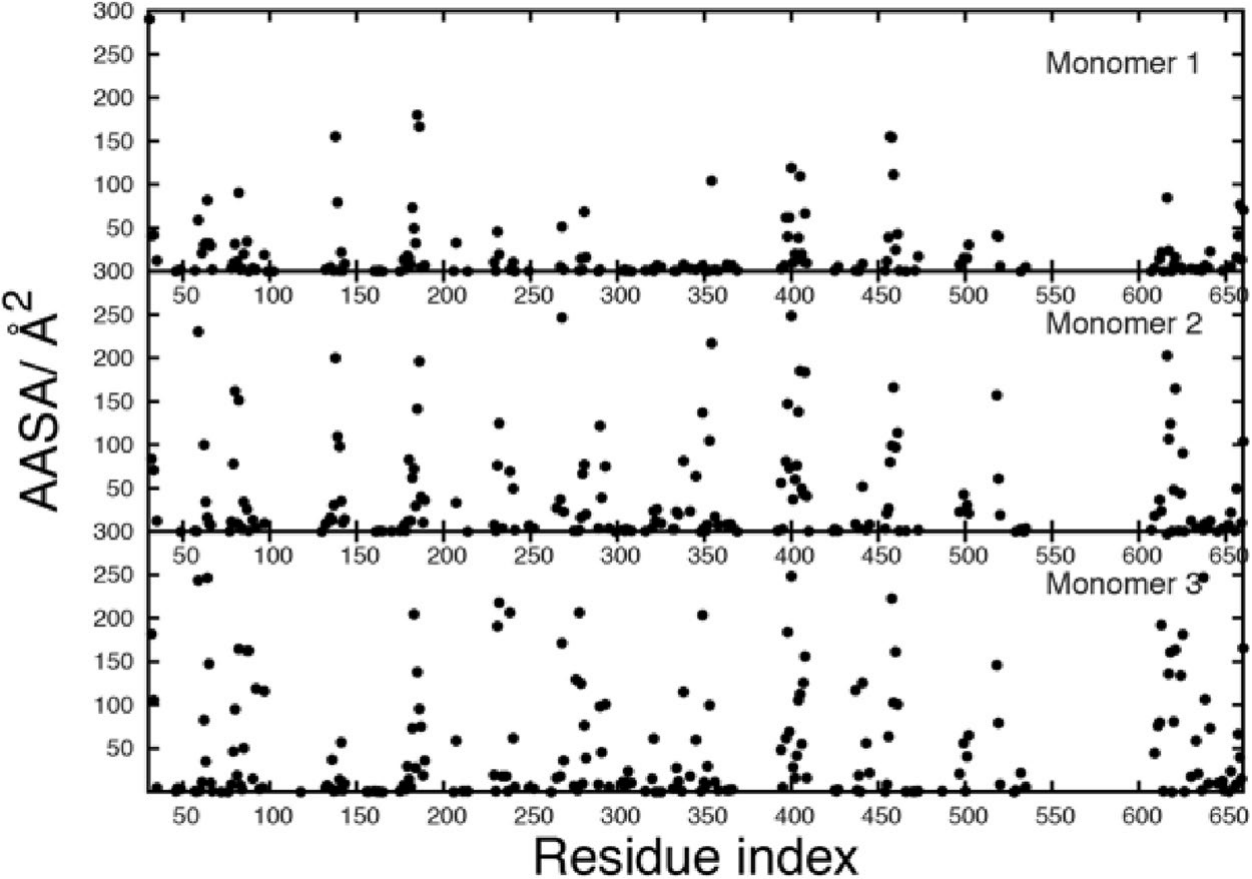
Average antibody accessible surface area (AASA), Å^2^, of the protein residues from the three monomers. Results are the average over the HREST-BP Env simulation sampled every 0.08 ns with AASA values calculated using a probe radius of 10 Å and an accuracy of 0.5. Values are presented for those residues with an average AASA of greater than 0.1 Å^2^.

Antibody and CD4 receptor binding with Env involves interactions with both protein and glycan moieties. Given the perceived role of the glycans as a shield that obstructs interaction with the protein surface, it is important to understand which regions of the Env protein are accessible to antibodies and CD4. This was performed by determining the average Antibody Accessible Surface Area (AASA) of the protein on a per amino acid residue basis over the entire Env simulation. The AASA was determined in a manner analogous to the solvent accessible surface area using a probe radius of 10 Å, which approximates an antibody combining region interacting with the protein^6, 15^. Results from the analysis are presented in Fig. 5. Evident are regions of the protein that are highly exposed versus those with little exposure, with the general pattern similar between the differentially glycosylated monomers. Amino acid residues aa138 to aa141 and aa177 to aa186, corresponding to the V1 and V2 loops, respectively, have large AASA values, specifically aa138-aa139 and aa182-aa186. In contrast, the V3 loop region from aa296-aa332 shows a number of residues to be exposed, though all with comparatively low AASA values. Omission of selected glycans in M2 and M3 leads to an increase in the AASA of regions of the V3 loop^55^. Residues that interact with CD4^55, 56^, including aa279-aa281, aa364-aa367, and aa456-aa460 show finite AASA values for multiple residues. Interestingly, in the known CD4 interacting regions that involve residues aa425-aa430 and aa469-aa474, only individual residues aa428 and aa474, respectively, have average AASA values > 1 Å^2^. These results indicate that CD4 likely interacts initially with small openings in the Env glycan shield, allowing for initial direct protein-protein interactions with subsequent conformational changes leading to more extensive protein-protein interactions. However, it should be noted that larger AASA values occur for the majority of residues at specific snapshots of the simulations (Figure S8), indicating that short-lived openings of the glycan shield may facilitate interactions of CD4 or antibodies with the protein.

### Impact of glycan holes on protein and glycan and protein epitope accessibility

Variability within the HIV Env glycosylation pattern is known to affect its ability to induce and become the target of bnAbs. This is in part due to the omission of specific Env glycans, a phenomenon referred to as glycan holes^43^. For example, aa241, which is a Ser in strain BG505.SOSIP.664 but an Asn in most HIV strains, produces a glycan hole leading to this residue being an important epitope for neutralizing Abs when BG505 is used as an immunogen^43^. In the present simulation, while aa241 has a small average AASA value of 0.1 Å^2^, the AASA of aa240 is 10.7 Å^2^ (Fig. 5) consistent with this region of the protein contributing to the immunogenicity due to the glycan hole caused by Ser241 in BG505^43^. Accordingly, the presence of glycan holes will impact the exposure of different aspects of Env to interaction with antibodies, thereby potentially influencing the generation of a humoral immune response capable of viral infectivity neutralization. To be able to systematically understand the potential impact of glycan holes, it is necessary to identify regions of both the glycan shield and the underlying protein that become accessible upon omission of specific glycans. The availability of comprehensive conformational distributions of the glycans and the underlying proteins generated in the present study allows for such analysis.

Impact of the omission of specific glycans was analyzed based on determination of changes in the average solvent accessible surface area (SASA) of the glycans or the AASA of the proteins. In the case of the glycans, the change in SASA was probed using a sphere the radius of water (1.4 Å) while the AASA values used a probe of 10 Å as discussed above. We note that the presence of a glycan hole will impact the conformational properties of the adjacent glycans and underlying protein, an effect that is not possible to take into account without performing additional HREST-BP simulations with the individual glycans removed, a computationally challenging task.

**Figure 6:**
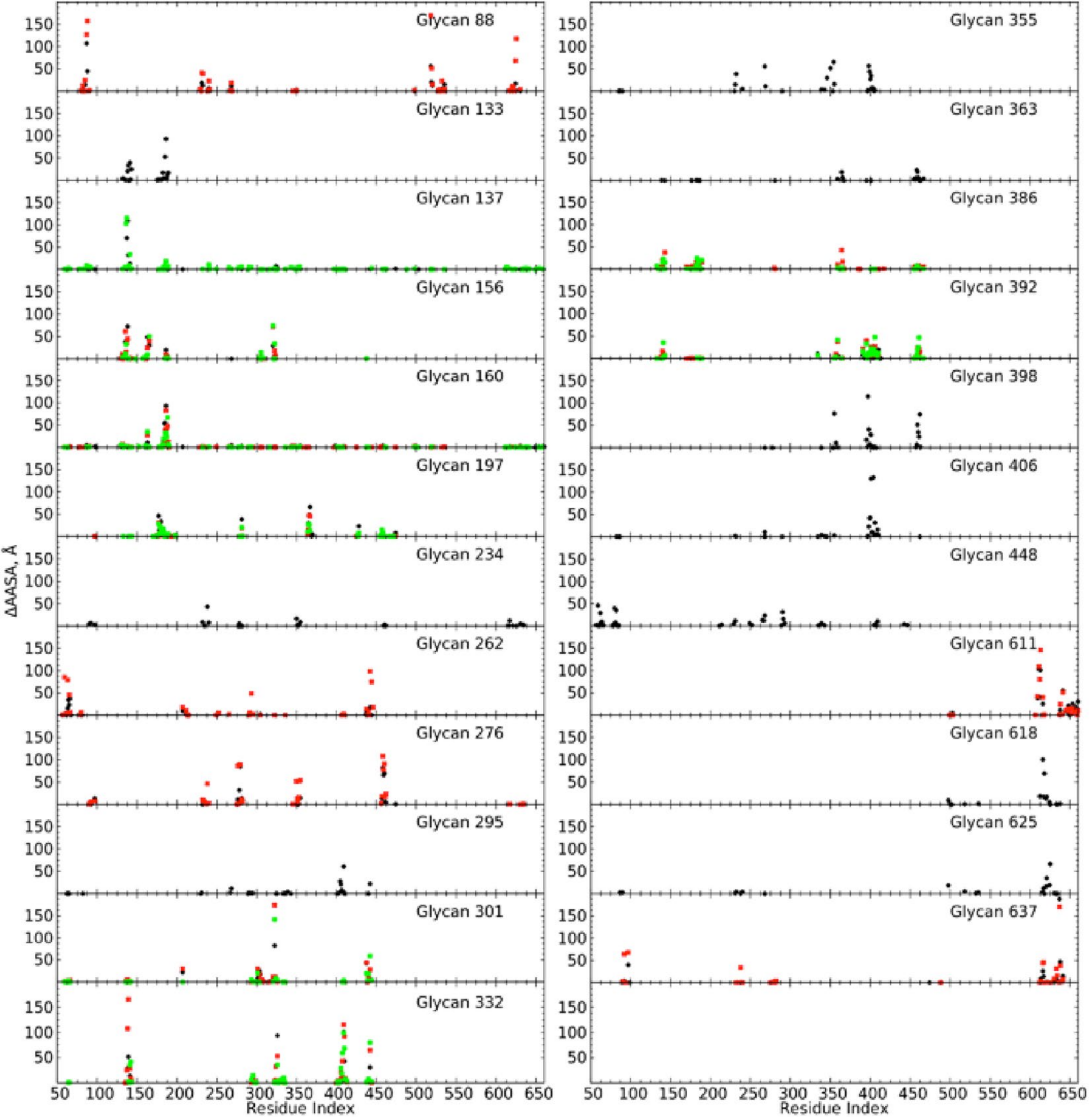
Change in antibody accessible surface area (ΔAASA, Å^2^) of all protein residues in the M1 gp120 and gp41 proteins for Monomer 1 (black spheres), Monomer 2 (red spheres) and Monomer 3 (green spheres). Only ΔAASA values greater than 0.1 Å^2^ are shown.

Results for glycans in which changes greater than 5 Å^2^ occurred are presented in Table 2 while plots of the change in all protein residues due to the glycan holes are shown in Fig. 6. In this analysis, glycan truncation involved the glycans on all three monomers of the trimer when present (eg. M1-g88, M2-g88 and M3-g88 were truncated when omitting g88). Analysis of Table 2 and Fig. 6 for glycans on monomer 1 shows as expected that the glycan holes lead to increased SASA of specific glycans and AASA of specific regions of the protein surface. As previously discussed^43^, such additional accessibility associated with the glycan holes may skew production of antibodies toward these available epitopes during the primary immune response, leading to neutralizing antibodies that may become ineffective for escape mutants that are glycosylated at the sites of holes in the original strains. In general, this may affect the capacity of bNAbs to recognize a broad range of cognate Env targets that present a diverse pattern of glycan substitution.

**Table 2:** Change in solvent accessible surface area (SASA, Å^2^) of glycans on monomer 1 due to the omission of selected glycans for which the change in SASA was >5 Å. Average values and standard errors (SE) are shown.

Glycan omitted	Glycan	Average difference	SE	Glycan omitted	Glycan	Average difference	SE
g88	g618	10.4	4.3	g363	g133	9.6	4.3
g88	g625	56.3	8.6	g363	g386	202.4	10.1
g133	g197	33.1	7.8	g363	g392	105.6	9.6
g133	g363	11.9	5.5	g363	g398	20.8	8.8
g133	g386	68.6	12.0	g386	g133	70.8	12.4
g137	g156	31.4	9.1	g386	g332	16.6	6.6
g137	g301	36.5	11.6	g386	g363	211.9	9.3
g137	g332	26.9	9.3	g386	g392	87.0	14.0
g156	g137	31.1	9.1	g392	g332	47.9	12.7
g197	g133	31.9	7.5	g392	g363	108.2	10.1
g234	g276	111.3	12.3	g392	g386	93.3	15.6
g234	g637	33.9	7.6	g392	g398	52.1	12.5
g262	g295	9.4	3.0	g392	g406	8.8	4.5
g262	g301	19.2	5.5	g398	g355	10.2	5.9
g262	g448	42.7	3.8	g398	g363	19.3	8.0
g276	g234	99.0	10.9	g398	g392	48.2	11.5
g276	g637	22.6	7.5	g398	g406	13.9	5.7
g295	g262	10.7	3.7	g406	g332	7.5	3.1
g295	g301	6.1	5.3	g406	g355	26.3	9.7
g295	g332	14.6	4.3	g406	g392	9.5	4.4
g295	g448	106.5	14.5	g406	g398	16.5	6.5
g301	g137	40.2	13.1	g406	g448	28.8	12.3
g301	g262	17.5	5.0	g448	g262	36.5	3.5
g301	g295	6.8	6.0	g448	g295	103.1	14.3
g332	g137	31.4	9.5	g448	g406	27.5	11.9
g332	g295	15.1	4.3	g611	g637	5.0	5.0
g332	g386	16.5	6.5	g618	g88	11.3	5.0
g332	g392	46.4	12.2	g618	g625	40.2	10.6
g332	g406	8.5	3.6	g625	g88	56.7	8.7
g355	g398	9.3	5.5	g625	g618	34.4	9.0
g355	g406	29.4	10.6	g637	g234	33.4	7.3
				g637	g276	25.3	8.4

Calculation of the change in SASA or AASA was performed by first determining the overall SASA or AASA for all atoms in the Env followed by determination of the SASA or AASA contribution of the individual glycans or protein residues, respectively. This procedure was then repeated with the overall SASA or AASA calculation performed with the individual, selected glycan omitted. The difference between the SASA or AASA values of the glycans or protein residues, respectively, between the truncated and full Env were then determined.

Alternatively, engineering of glycan holes may have potential for facilitating vaccine design. Broadly neutralizing antibody PG9 primarily interacts with g160 with additional interactions with g156. The antibody also has interactions with amino acid residues 167 to 173 in the V1/V2 loop. Analysis of Fig. 6 shows that omission of g156 leads to improved accessibility of residues aa163 and aa165, potentially allowing improved interactions between the complementarity determining region (CDR) H3 of PG9 and the gp120 protein. Interestingly, M1-g160 has a relatively small number of interacting partners (6) (Table 1). The extent of overlap with those partners, based on the OC analysis (Table S6), is small, being 0.012 (with M1-g156) or less excluding the value of 0.022 with the corresponding glycan on monomer 3, M3-g160. These results suggest that the inherent exposure of M1-g160 contributes to its role as an antibody epitope and that these interactions may potentially be enhanced by a glycan hole generated via omission of g156. In addition, omission of g156 would increase exposure of protein residues on the V1/V2 loop thereby creating a better immunogen. Consistent with this are experimental studies on a scaffolded V1/2 construct showing that the presence of g156 leads to a 2-fold decrease in affinity for PG9^57^.

The PGT121 family of antibodies interact with the Env in the region of g332, which acts as the essential epitope for multiple antibodies. Glycan g137 is also known to contribute to the epitope^6, 15, 17^. However, in contrast to g160, both g137 and g332 have a high number of interacting partners (11, Table 1). A number of the interacting glycans have OC values of 0.17 or more with g332 including M1-g137, M1-g295, M1-g301, M1-g386, M1-g392 and M1-g406. Glycan g137 has large OC values with M1-g301 and M1-g156. Glycan g156 is also known to be protected from endoglycosidase hydrolysis by PGT122^17^; in the present simulation contact between g332 and g156 is blocked by g137 leading to the OC between g332 and g156 being zero. Given the close spatial relationship of these glycans, their omission leads to greater SASA exposure of g332 (Table 2) with the largest effect observed with M1-g392 (ΔSASA = 48 ± 12Å^2^). PGT121 family antibodies also have direct interactions with residues aa325 to aa328 in the V3 and residues aa135 to aa139 in the V1 loops of gp120. Only omission of M1-g332 itself leads to substantial additional exposure of aa323 and aa325, with omission of g137 and g301 leading to small increases in the AASA of these residues. Of residues aa135-aa139 in the V1 loop only g332 and g156, besides g137, lead to large increases in their AASA. Such small differences in AASA indicate the challenge of making this protein epitope more accessible for antibody design even with the capability of selective creation of glycan holes.

### Antibody or CD4-Env Glycan Interactions

To better understand the implications of the conformational heterogeneity of the Env glycans identified in the present study with respect to vaccine development strategies, we undertook analysis of the glycans with the potential to have direct interactions with selected antibodies or the CD4 receptor when bound to Env. Analysis was performed by first modeling the orientation of each antibody on snapshots from the Env HREST-BP simulation as described in Section S.5 of the supporting information and calculating minimum distances between each Abs and all glycans or the volume occupied by the Abs. Antibodies that were modeled include PG9, 17B, PGT135, 35O22 (2x), PGT122 (2x), 8ANC195 and VRC01, as well as the CD4 receptor (Table 3). Antibodies were selected as they are representative of the different well-studied classes targeting the Env. PGT122 and 35O22 were analyzed twice as they were on two of the crystal structures selected to cover the full range of classes of antibodies^6, 15^. The minimum distance between the non-hydrogen atoms of each glycan and antibody or the CDR regions of the antibodies was then determined over the course of the simulation, with analysis based on cutoff distances of 10 Å and 3 Å, respectively. The number of glycans coming within these distances of the antibodies is shown in Table 3, with the identity of each glycan coming within 10 Å of the antibodies or CD4 presented in Table S7a and b. The cutoff distance of 10 Å was selected to account for the lack of flexibility in the antibodies due to the rigid modeling approach. From the 10 Å cutoff analysis, it can be seen that the antibodies interact with 7 to 14 glycans and the CD4 receptor predicted to interact with 13 glycans. When only the CDR regions of the antibodies are considered, the number of glycans typically decreases by one versus the value with the full antibody. Thus, the present results indicate that 7 or more glycans can potentially interact with each of the studied antibodies.

**Table 3:** Number of glycans that approach within 10 or 3 Å of the modeled antibodies for the entire antibody and for all of the complementary-determining regions (CDR) of each antibody.

PDB/Antibody	10 Å cutoff		3 Å cutoff	
	Full Antibody	All CDRs	Full Antibody	All CDRs
PG9 (3U4E)^58^	7	7	7	7
a17b (4JM2)^16^	14	13	11	8
CD4 receptor (4JM2)^16^	13	NA	9	NA
PGT135 (4JM2)^16^	12	11	8	8
35O22 (4TVP)^15^	8	7	6	4
PGT122 (4TVP)^15^	12	11	8	7
35O22 (5FYJ)^6^	10	7	6	5
PGT122 (5FYJ)^6^	12	11	9	9
VRC01 (5FYJ)^6^	14	11	8	6
8ANC195 (5C7K)^14^	9	8	8	8

Analysis similar to that in Table 3 was presented previously based on standard MD simulations of M5, M7 and M9 glycosylated Env trimers based on a 3 Å cutoff (see Figure 6 of ref. 6). In that study, the antibodies were shown to interact with 1 or 2 essential glycans; our analysis is consistent with those results with respect to the number and identity of the essential glycans (Table S7). However, the previous study indicates the number of non-essential glycans to range from 1 to 9 glycans. In contrast, our analysis indicates larger number of interacting glycans. For example, in the previous study PG9 was indicated to interact with 4 or 5 glycans, compared with 7 in the present study. Similar trends occur with PGT122 with 2 versus 8 or 9 in the present study, 35O22 with 4 to 6 in the previous study versus 6 presently and 4 to 6 with VRC01 in the previous study versus 8 in the present study. The increased number of interacting glycans emphasizes the advantage of the enhanced sampling method used presently versus standard MD alone and further indicates how the conformational heterogeneity of the glycans will potentially impact glycan-antibody interactions.

Additional analysis based on the OC values of the glycan and antibody or CD4 receptor 3D Cartesian volumes (Table S8) were consistent with the minimum distance analysis in Table S7. The analysis was based on 375 evenly distributed snapshots from the HREST-BP ground-state replica. OC analysis that just considered the antibody CDRs also showed a large number of glycans to interact with those regions (Table S9). These results further indicate that an extensive number of glycans have the potential to interact with the individual antibodies.

Results in Tables S7, S8 and S9 can be interpreted in the context of specific antibodies, building on the information obtained for glycan-antibody interactions detailed in the 2016 crystallographic study supplemented with MD simulations (see Fig. 6 of that study)^6^ as well as other experimental studies. For PG9, g156 and g160 have been indicated to be essential glycans and g160 from the other protomer subunits can interact with PG9 on a given protomer, which defines the quaternary epitope. Besides g156 and g160 identified from experimental studies^57–59^, g137 has been indicated to interact with PG9. The present results indicate considerable interactions with g137 as well as with g197 and g301 based on both the OC and minimum distance analyses. For antibodies PGT122 and 35O22, OC values for the two rebuilt models using crystal structures 4TVP^15^ and 5FYJ^6^ are consistent with each other. Essential glycans for PG122 include g137 and g332 and only g392 was previously indicated to also interact with that antibody^6^. However, the present results indicate a large number of antibodies to interact, including g301, g156, g133 and g386 as well as g392. Of the 3 glycans (g137, g301 and g332) with significant overlap with PGT122, the OC value of g301 on monomer 3 (M3) is much larger than that on M1 and M2. This further suggests the local environment can affect the conformational preference of the glycans, as the glycans on M3 are the most dispersed. Glycan 88 is required for 35O22 and g234, g355, g618 and g625 were observed interact with that antibody. Our results are consistent with the experimental observations with additional interactions with g611, g637 and g227 occurring. Notably, these interactions often involve the CDR regions of the antibodies. Glycans g332 and g392 are required for PGT135. The present results also show substantial interactions with g133, g137, g197, g301, g332, g363, g386, g398, and g406, again a much higher number of interactions than observed experimentally. The trend of increased numbers of glycans potentially interacting with each antibody over that observed in the previous study is present for 8ANC195 and VCR01. For 8ANC195 the essential glycans g234 and g276 all have considerable overlap as do g88, g355, g398, g611, g618, c625 and g637. A large number of glycans interact with VCR01 and the CD4 receptor with the identity of those glycans, including g234, g276, g363 and g386, having potential interactions with both species. This analysis further shows the large number of glycans that can potentially interact with the antibodies and CD4, with that number being substantially larger than that observed in previous studies.

### Glycan conformations that maximize or minimize interactions with antibodies

As the present computational study identified a substantial number of previously unidentified antibody-glycan interactions it could yield insights into maximizing or minimizing such interactions, information that could be used to engineer candidate vaccine constructs. To systematically investigate this, the GL conformations of non-essential glycans that have direct interactions with the CDR regions of the antibodies were identified based on the data in Tables S7b and S9. The initial analysis involved the five linkages in the GL cluster definitions that include the protein χ1/χ2 dihedrals, φ_s_/ψ_s_ values, and the three linkages in the unbranched region of the glycan as these linkages will dominate the overall orientation of the glycan over the protein surface. From the analysis, several possible such scenarios as listed in Table 4 were identified. With M1-g137 and PG9, GL cluster 31223 of g137 is sampled with a 19% probability, with the majority of those conformations being within 5 Å of PG9. Accordingly, stabilizing the glycan in this conformation would maximize glycan-antibody interactions. Alternatively, stabilizing the 34223 conformation would limit the number of glycan-antibody interactions. Similar results are observed with other glycan-antibody pairs. Stabilizing M1-g406 in either the 11223 or 10223 GL cluster conformation would minimize glycan-PGT135 interactions. With M1-g137 stabilization of the 31233 conformation would maximize interactions with the 17b antibody while stabilizing 11223, 34223 or 14223 would minimize glycan-antibody interactions. Stabilization of GL conformations 30223 or 31223 of M1-g625 would minimize interactions with 8ANC195 whereas stabilization of 15223 is predicted to maximize glycan-antibody interactions. While stabilization of specific glycan conformations would be challenging, potential scenarios involving chemical modification of glycans and/or engineering of specific protein-glycan interactions may achieve such an outcome, representing an extension of structure-based vaccine design focused on protein alone^17, 60^. We also note that controlling glycan conformations may also be of utility to maximize or minimize the accessibility of the protein surface. Importantly, results from molecular simulations may act as a guide for such efforts that could be combined with multivalent approaches involving multiple modified Env constructs to obtain broad coverage vaccine candidates.

**Table 4:** Probability of sampling GL clusters based on the first 5 linkages for all model snapshots and those with the minimum distances >or <5 Ang for selected glycans with the CDR regions of selected glycans. GL cluster Probability.

GL cluster	Probability		
	Total	<5 Å	>5 Å
M1-g137-PG9 (3U4E)			
11223	0.21	0.09	0.13
31223	0.19	0.17	0.03
34223	0.16	0.01	0.15
14223	0.13	0.02	0.11
10223	0.07	0.03	0.04
35123	0.06	0.00	0.06
M1-g406-PGT135 (4JM2)			
11223	0.41	0.10	0.31
10223	0.17	0.02	0.15
15223	0.11	0.06	0.05
31223	0.10	0.00	0.10
14223	0.07	0.04	0.03
M1-g137-17b (4JM2)			
11223	0.21	0.06	0.15
31223	0.19	0.17	0.03
34223	0.16	0.00	0.16
14223	0.13	0.00	0.13
10223	0.07	0.02	0.05
35123	0.06	0.01	0.05
M1-g625-8ANC195 (5C7K)			
30223	0.33	0.00	0.33
31223	0.26	0.01	0.26
15223	0.16	0.13	0.03
34223	0.07	0.06	0.01
14223	0.06	0.06	0.00

Only GL clusters sampled at a probability of 0.05 or more for either <or >5 Å are shown.

## Conclusions

Molecular simulations at ambient temperatures using enhanced sampling technologies in combination with GPU computing are shown to produce an extensive atomistic representation of the structure and dynamics of the glycan shield of the HIV envelope. The range of glycan conformational sampling, including glycan-glycan and glycan-antibody interactions, is predicted to be more extensive than previously observed in experimental studies. Regions of the underlying Env protein exposed through the glycan shield are identified, indicating that small openings in the glycan shield allow for initial interactions with antibodies or the CD4 receptor followed by conformational changes in the local glycans yielding the final antibody-Env complex stabilized glycan interactions. Detailed analysis of the conformational sampling of glycans based on GL clusters indicates that preorganization of the glycans contributes favorably to antibody binding. Modeling of known antibodies onto the Env simulation system identifies a more extensive network of glycan-antibody interactions than that observed experimentally. Notably, results from the simulations can be used to predict the impact of glycan holes on both glycan and protein accessibility, and identify specific glycan conformations that will i) maximize or minimize interactions with antibodies or ii) maximize accessibility of regions of the protein surface in order to facilitate interactions with antibodies. Overall, it is evident that the high information content accessible from the present Env simulation as well as future studies on specific Env constructs will be of utility in the design and engineering of candidate HIV vaccines that include the Env protein.

## Methods

Simulation studies were undertaken on an HIV envelope glycoprotein (Env) trimer model constructed on the basis of the pre-fusion crystal structure of BG505 SOSIP·664 (PDBID: 4TVP) (see supporting information)^15^. The Env was differentially glycosylated on each monomer with M5 and M9 glycans at selected sites (Table S1 and Figure S1, supporting information). Four additional systems studied include one M5 glycan covalently connected with an Asn dipeptide, one M5 linked to a reduced V1V2 region of gp120 (PDBID: 4DQO)^59^, one M9 glycan bound to an Asn dipeptide and one M9 glycan bound to the gp120 core structure (PDBID: 4R2G)^20^. A detailed description of the setup of the systems is included in the supporting information with specific details listed in Tables S1 and S2.

To enhance the sampling of the N-glycan conformations beyond that obtained from standard MD simulations we implemented our HREST-BP method^33, 34^ into OpenMM^61^ and linked to the CHARMM program^62^, allowing the simulations to be performed with GPU acceleration. The scaled potential for the m-th replica is expressed as,

1 $$\begin{array}{ccc}{U}_{m}({\boldsymbol{R}}) & = & \frac{{\beta }_{m}}{{\beta }_{0}}({U}_{s}^{short}({{\boldsymbol{R}}}_{s})+{\lambda }_{m}{V}_{b}({\rm{\Omega }}({{\boldsymbol{R}}}_{s})))+\sqrt{\frac{{\beta }_{m}}{{\beta }_{0}}}{U}_{se}^{short}({{\boldsymbol{R}}}_{s},{{\boldsymbol{R}}}_{e})\\  &  & +{U}_{s}^{long}({{\boldsymbol{R}}}_{s})+{U}_{se}^{long}({{\boldsymbol{R}}}_{s},{{\boldsymbol{R}}}_{e})+{U}_{e}({{\boldsymbol{R}}}_{e})\end{array}$$

where the conformational space of the whole system ***R*** is decomposed into two subspaces represented by the central subsystem ***R***_s_ and environment ***R***_e_, resulting in three interaction components, the internal energy of central subsystem ${U}_{s}^{short}({{\boldsymbol{R}}}_{s})+{U}_{s}^{long}({{\boldsymbol{R}}}_{s})$, the self-interaction energy of environment U_e_(***R***_e_), and the interaction energy between the central subsystem and environment ${U}_{se}^{short}({{\boldsymbol{R}}}_{s},{{\boldsymbol{R}}}_{e})+{U}_{se}^{long}({{\boldsymbol{R}}}_{s},{{\boldsymbol{R}}}_{e})\cdot {V}_{b}({\rm{\Omega }}({{\boldsymbol{R}}}_{s}))$ is the biasing potential applied to a set of collective variables, Ω(***R***_*s*_), in the central subsystem and *λ*_*m*_ is the scaling factor for the biasing potential with positive values for non-ground-state (ie. excited-state) replicas. *β*_*0*_ is the inverse temperature (*β*_*0*_ = 1/*k*_*b*_ *T*_*0*_) at which the simulation is going to be performed and *β*_*m*_ the inverse temperature (*β*_*m*_ = 1/*k*_*b*_ *T*_*m*_) used to scale the short-range potential energy of central subsystem and interaction energy between the two subsystems at replica *m*.

In this CHARMM-OpenMM implementation of HREST-BP, we define the “central subsystem” or solute as the N-glycans in the respective simulation systems, as these were subjected to enhanced sampling. Enhanced sampling was achieved by subjecting the glycosidic linkages to potential biasing with concurrent effective temperature scaling of the intra-solute potential and solute-environment interactions. The biasing potentials *V*_*b*_(Ω(***R***_*s*_)) were formulated with the 2-dimensional grid based correction map (bpCMAP) along the torsional angles of φ_s_/ψ_s_, χ_1_/χ_2_ and all 1→6 glycosidic linkages in M5 or M9 glycans and were constructed using the corresponding disaccharide model in the gas phase as described previously^33, 34^. In 1 → 6 glycosidic linkages, the bpCMAPs were only applied to bias the movement about ψ and ω since the motion along torsional angle φ is relatively restricted with respect to the dihedrals ψ and ω^33, 34^. Biasing potentials were not applied to 1→2, 1→3 and 1→4 linkages to reduce the number of replicas. In contrast to the previous implementation of HREST-BP with an exact partition of interactions^33, 34^, the long-range part of electrostatic interaction, including those between the central subsystem and images of central subsystem ${U}_{s}^{long}({{\boldsymbol{R}}}_{s})$, and the central subsystem and images of environment ${U}_{se}^{long}({{\boldsymbol{R}}}_{s},{{\boldsymbol{R}}}_{e})$ calculated using particle Mesh Ewald, together with all the environment-environment interactions U_e_(***R***_e_), were included in the unperturbed term in this CHARMM-OpenMM version. This is because the separation of long-range electrostatic interactions between different parts of the simulation system is not possible in the OpenMM version being used. However, the local, real space electrostatic interactions were subjected to solute-tempering, thereby facilitating sampling. In the HREST-BP simulations, an exchange attempt was examined every 2000 MD steps according to the Metropolis criterion. The ground-state replicas were simulated unperturbed at 298 K and the temperatures of other replicas were determined with a geometric distribution. The HREST-BP simulations were carried out for all systems. The number of replicas and corresponding inverse temperature (β_m_) and scaling factor (λ_m_) distributions were determined according to the system size, with the simulation parameters presented in Table S3. Using these parameters a successful exchange (or acceptance) ratio of 18% or larger was obtained for any neighboring replicas (Table S3)^34^. Before the HREST-BP production run, the Env trimer system was subjected to standard MD in which it was heated from 100 K to 200 K to 298 K with gradually reduced restraints applied to protein and glycan non-hydrogen atoms under the NPT ensemble using NAMD package^63^. Then the system with all restraints removed was equilibrated with a 10 ns standard MD simulation in the NVT ensemble. 300 ns HREST-BP simulations were performed for the five systems in the production stage. With the GPU acceleration, we obtained approximately 3.4 ns/day for each replica of the Env trimer on one GeForce GTX 980 card. Analysis of the replica random walks (Figure S7) shows the replicas to all sample a range of replica space, indicating that extensive sampling was occurring in the simulations. In addition to the HREST-BP simulations, a standard MD simulation was performed for the Env trimer model for 400 ns. The improvement in sampling associated with the HREST-BP method over standard MD along with convergence of the HREST-BP simulations are discussed in section S.6 of the supporting information.

All simulations were performed under the CHARMM36 additive force field for proteins^64, 65^ and carbohydrates^37, 40, 66^. The temperature was maintained at 298 K using the Hoover algorithm with a thermal piston mass of 1000 kcal/mol·ps^2^ ^67^ and a constant pressure of 1 atm was realized using the Langevin piston algorithm with a collision frequency of 20 ps^−1^ and mass of 1630 amu^68^. The covalent bonds involving hydrogens were constrained with SHAKE, which allows an integration step of 2 fs in the MD simulations^69^. In the energy and force evaluations, the nonbonded Lennard-Jones interactions were computed with a cutoff of 10 Å with a switching function applied over the range from 8 to 10 Å. The use of cutoff value of 10 Å produces almost the same probability distribution along glycosidic linkages as compared to the typical value of 12 Å used in other studies while increasing the speed of the simulation by 30% for a system with 42,000 atoms (data not shown)^34^. The electrostatic interactions were treated by the particle mesh Ewald method with a real space cutoff of 10 Å, a charge grid of 1 Å, a kappa of 0.34, and the 6-th order spline function for mesh interpolation^70^.

## Electronic supplementary material

Supporting information
