# Supplementary material for: Conformational Heterogeneity of the HIV Envelope Glycan Shield
Source: Sci Rep. 2017 Jun 30;7:4435. doi: 10.1038/s41598-017-04532-9 (PMC5493700; doi:10.1038/s41598-017-04532-9)
Supplement: Supplementary file 1 — Supporting information [file 41598_2017_4532_MOESM1_ESM.pdf]

Supporting Information

Conformational Heterogeneity of the HIV Envelope Glycan Shield

Mingjun Yang,[1] Jing Huang,[1] Raphael Simon,[2] Lai-Xi Wang[3] and Alexander D. MacKerell, Jr.[1,*]

[1]Department of Pharmaceutical Sciences, School of Pharmacy, University of Maryland, Baltimore, Maryland, USA

[2]Center for Vaccine Development, Institute for Global Health, School of Medicine, University of Maryland, Baltimore, Maryland, USA

[3]Department of Chemistry and Biochemistry, University of Maryland, 8051 Regents Drive, Room 3500, College Park, Maryland, USA

*Corresponding author. amackerell@rx.umaryland.edu, 410-706-7442

S.1) Model construction

The HIV envelope glycoprotein (Env) trimer model was constructed on the basis of the pre-fusion crystal structure of BG505 SOSIP·664 (PDBID: 4TVP).[1] This trimer contains three gp120 and gp41 subunits, respectively. In this study, we adopted the residue numbers of the HxB2 system.[2] The undetermined residues in gp120 (residues 1-30, 185A-185I, 400-410 and 506-513) were modelled with the SWISS model tool,[3] while the missing loops in GP41 (residues 512-517 and 548-568) were not completed due to potential spatial overlap with other residues determined in the structure. The termini surrounding these regions were capped with methylated peptide moieties. The protonation states of the titratable protein residues were examined with the pKa prediction tool ProPka and all these residues were set to standard states.[4] The possible flips of all the Asn, Gln and His residues were checked and adjusted with MolProbity.[5] All the disulfide bonds within gp120 or between gp120 and gp41 were included in the simulation models. All crystal waters were preserved and the two antibodies PGT122 and 35O22 were removed from the model.[1] The Env trimer model was built with the three monomers glycosylated in crowded and dispersed conditions, that is, one monomer with 23 sites glycosylated and the other two with a reduced number of 12 and 8 sites glycosylated, respectively (Table S1). In the two monomers with reduced glycosylation, only glycans corresponding to important epitopes and the ones close to the interface of the fully glycosylated protomer were included. In such a model, the conformational heterogeneity of individual glycans can be extracted under different glycosylation states from only one simulation and the system size is reduced by ~120,000 atoms compared to a model with three fully glycosylated monomers, thereby saving computational burden.

For each site, the high mannose saccharides M5 or M9 (Figure S1) were covalently linked to the amide side chain of the corresponding Asn residues (Table S1). In the Env trimer the fully glycosylated monomer is designated M1, the partially glycosylated monomer with 12 glycans is designated M2 and the third partially glycosylated monomer is designated M3. Thus, glycans on protomer M1 have the most crowded environment and those on M3 are in the most dispersed condition. Accordingly, glycans in the different monomers will be indicated based on their monomer ID, a "g" followed by the residue number to which they are linked (eg. M1-g88, M2-g88 and M3-g88). Selected glycosylated Asn residues were based on several representative crystal structures (Table S1),[1,6,7,8,9,10,11,12] with most identified as partial or full putative antibody binding epitopes.[12] The sites N332 and N392 are glycosylated with the high mannose glycan M9 since these two sites are involved in the interaction with the broadly neutralizing antibodies PGT122, PGT124, PGT128 and PGT135, as is shown by the corresponding antibody-antigen crystal structures.[1,8,10,12] All other sites in the computational model were glycosylated with M5.[13] The conformations of the M5 and M9 glycans were initially built by overlaying the crystal structure that has the N-glycan with the number of monosaccharides closest to that of the full M5 or M9 glycans linked to the corresponding Asn residues in 4TVP,[1] using that glycan conformation and building the remaining monosaccharides based on the internal coordinates specified in the additive CHARMM carbohydrate force field.[1,14,15,16,17] It is noted that two sites N339 and N462 are not glycosylated in the simulation models because the glycan stem (the two Glc*p*NAc residues) does not support the full mannose branch in M5 and clashes with protein residues. A cubic TIP3P water[18,19,20] box was constructed with a size of

150Å×150Å×150Å to solvate the Env model. A total of 18 chloride ions were added to neutralize the solvated systems. The final system contains 358,836 atoms.

Besides the Env trimer model, four other systems were constructed and simulated. These included one M5 glycan covalently connected with an Asn dipeptide, one M5 linked to a reduced V1V2 region of gp120 (PDBID: 4DQO),[7] one M9 glycan bound to an Asn dipeptide and one M9 linked to the gp120 core structure (PDBID: 4R2G).[12] The two reduced gp120 models were prepared as described above for the Env model including the missing loop construction, protonation state determination, and residue flip examination. These four models were also solvated with the TIP3P water box and neutralized with sodium or chloride counterions. Details of these systems are described in Table S2 of the supporting information.

Table S1. The glycosylated Asn residues and corresponding N-glycans included in the computational model of the Env trimer.

Residue number[a]	Monomer1	Monomer2	Monomer3
88 (88)	M5	M5	
133(133)	M5		
137(137)	M5		M5
156(148)	M5	M5	M5
160(152)	M5	M5	M5
197(197)	M5	M5	M5
234(234)	M5		
262(262)	M5	M5	
276(276)	M5	M5	
295(295)	M5		
301(301)	M5	M5	M5
332(331)	M9	M9	M9
355(354)	M5		
363(362)	M5		
386(385)	M5	M5	M5
392(391)	M9	M9	M9
398(397)	M5		
406(404)	M5		
448(446)	M5		

611(611)	M5	M5
618(618)	M5	
625(625)	M5	
637(637)	M5	M5

a. The initial numbers correspond to the Asn residue numbers in the crystal structure and are the numbers used in the text. The numbers in parenthesis are those counted sequentially in the computational model.

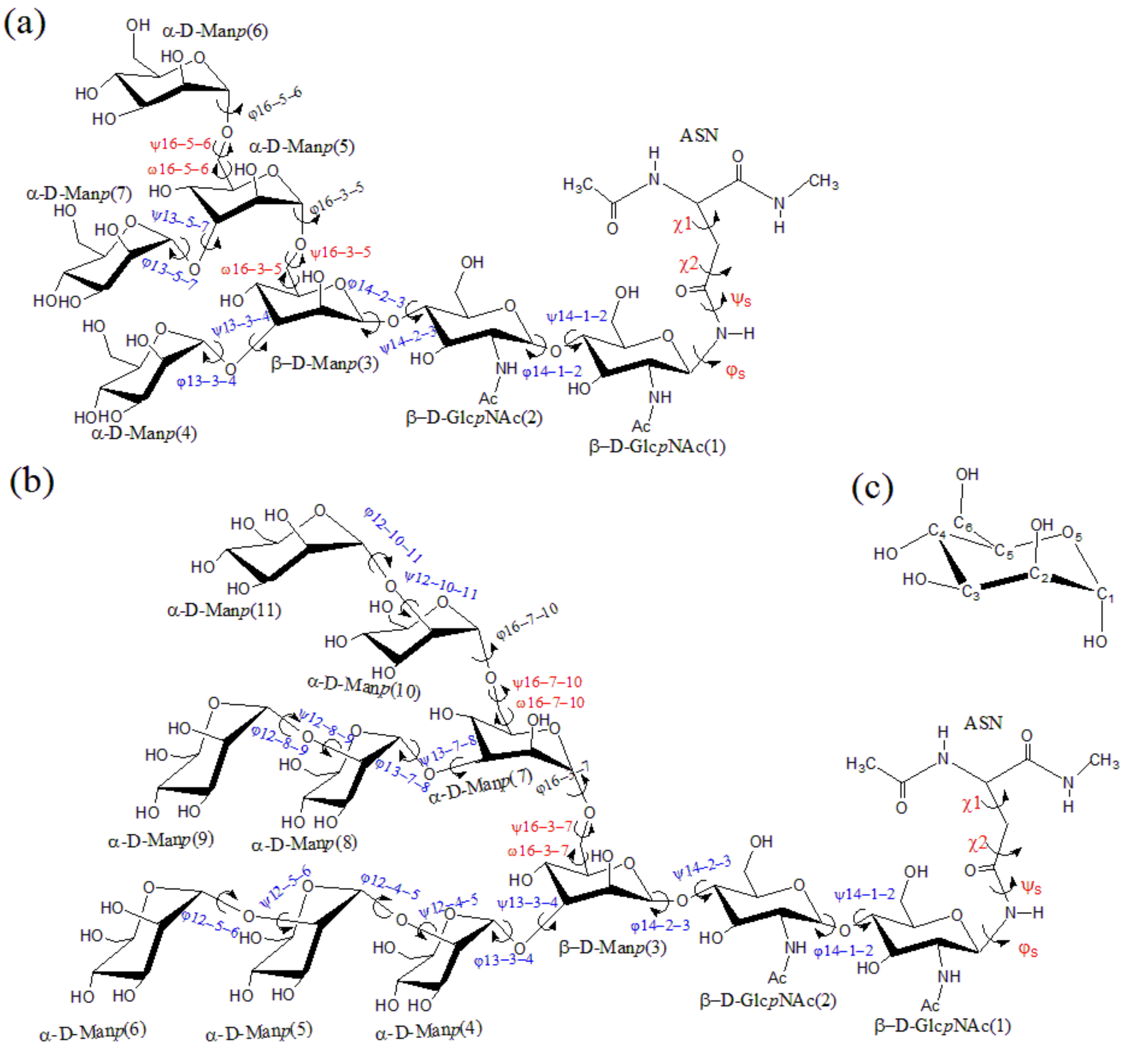

Figure S1. Structural model of (a) M5 and (b) M9 glycans connected to Asn dipeptide including the individual monosaccharide names along with identifiers of the dihedrals associated with the glycosidic linkages. (c) Atom name of the atoms in the monosaccharide. The aliphatic hydrogens were omitted for clarity. The dihedral definitions are φ(O_5'-C_1'-O_n-C_n)/ψ(C_1'-O_n-C_n-C_{n+1}) for *1→n* (n=2, 3 or 4) linkages, φ(O_5'-C_1'-O_6-C_6)/ψ(C_1'-O_6-C_6-C_5)/ω(O_6-C_6-C_5-O_5) for *1→6* linkages, χ1(N-CA-CB-CG)/χ2(CA-CB-CG-ND2), ψ_s(CB-CG-ND2-C1)/φ_s(CG-ND2-C1-O5). The two Glc*p*NAc residues are defined as the stem of the glycan and the mannose residues composes of the glycan branch. In the dihedral angle labels, the numbers, xx-y-z, follow φ, ψ, or ω, e.g. for φ14-1-2 where xx=14, y=1, z=2, represent the type of glycosidic linkage (xx), and the indices of the reducing- (y) and non-reducing-end (z) monosaccharides, respectively.

Table S2. Details of the studied Glycan systems.

System	Env trimer	M9-gp120 core	M5-gp120 v1v2	M9	M5
Number of atoms	360K	75K	49K	17K	14K
Number of protein residues	1797	308	115	1	1
Cubic box length/Å	150	90	79	55	51
No. of counter ions	18 Cl^-	5 Cl^-	3 Na^+	0	0

Table S3. The temperature (T) and scaling factor (λ) distribution and the average acceptance ratio (AR) in HREST-BP simulations of different systems.

replica	Env Trimer			M9-gp120 core[a]			M5-gp120 v1v2[a]	M9[a]	M5[a]
	T/K	λ	AR/%	T/K	λ	AR/%	AR/%	AR/%	AR/%
1	298	0.00	25.2	298	0.00	32.7	43.3	32.6	17.2
2	302	0.04	25.2	319	0.10	32.7	43.3	32.6	17.2
3	307	0.08	24.4	341	0.20	33.7	44.1	27.7	15.6
4	311	0.12	24.4	366	0.30	33.8	44.7	30.6	23.0
5	316	0.16	24.3	392	0.40	33.4	44.8	29.5	23.5
6	320	0.20	23.5	420	0.50	31.8	45.0	29.3	17.9
7	325	0.24	22.2						
8	330	0.28	22.6						
9	334	0.32	23.1						
10	339	0.36	22.7						
11	344	0.40	21.8						
12	349	0.44	20.6						
13	354	0.48	20.1						
14	359	0.52	19.2						
15	364	0.56	19.4						
16	370	0.60	18.6						

a. The same temperature and scaling factor distributions were used in the M9-gp120 core, M5-gp120 v1v2, M9, and M5 HREST-BP simulations.

S.2 Glycosidic linkage based description of N-glycan conformations (GL clusters)

From the simulations, clustering analysis was performed on the basis of the glycosidic linkages (GL) to characterize the conformational heterogeneity or preference in each saccharide molecule as in previous studies.[21] Each GL cluster or conformation can be represented based on torsion angles φ_s/ψ_s, χ_1/χ_2, ϕ/ψ for 1→2, 1→3 and 1→4 linkages, and ψ/ω for 1→6 glycosidic linkages. Figure S2 of the supporting information shows the

partitioning of the 2D dihedral space corresponding to different local minima on the corresponding free energy landscapes calculated as described below in Section S.3. Each partitioned region is then assigned an integer identifier. Therefore, GL clusters provide a convenient high-dimensional representation of the carbohydrate conformation. As previously discussed, as each GL Cluster represents a specific set of glycosidic linkage local minima, conformational variability within a GL cluster will not encounter high energy barriers, while conformations in different GL clusters are separated by one or more barriers associated with protein $\chi1/\chi2$, φ_s/ψ_s or glycosidic linkage dihedrals. To simplify the analysis, results are presented as 12 GL cluster integers for M9 (ie. 123456789012) while 8 are present for M5, where linkages not present in M5 are indicated as N (ie. 12345NN89N1N), as shown in Table S4 of the supporting information. Recorded snapshots saved every 8 ps from the ground state replica were used for GL clustering.

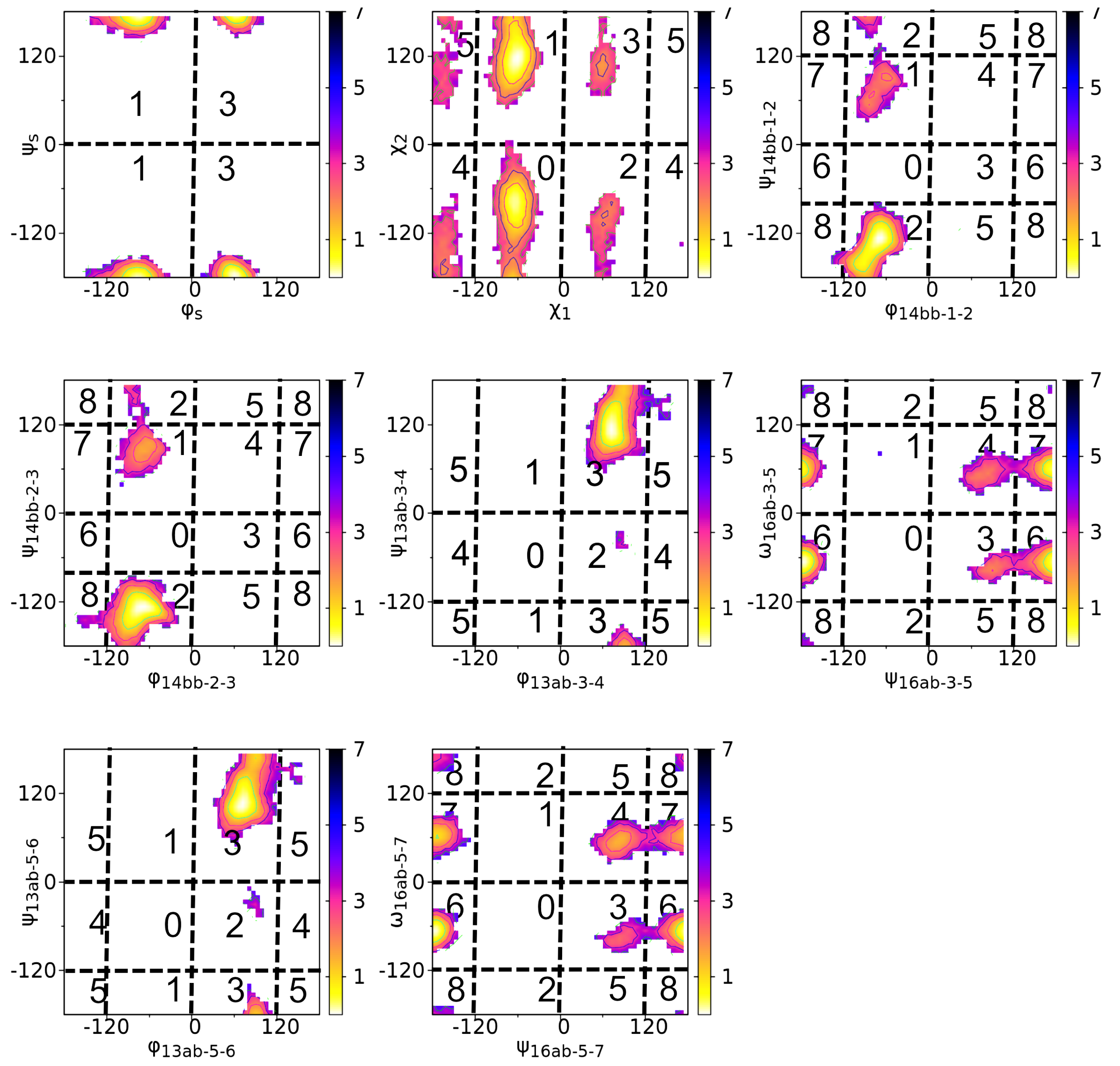

Figure S2a. Definitions of the integer values of the GL clusters for the M5 Glycan overlaid on 2D PMFs obtained from the M5 glycan with Asn dipeptide simulation.

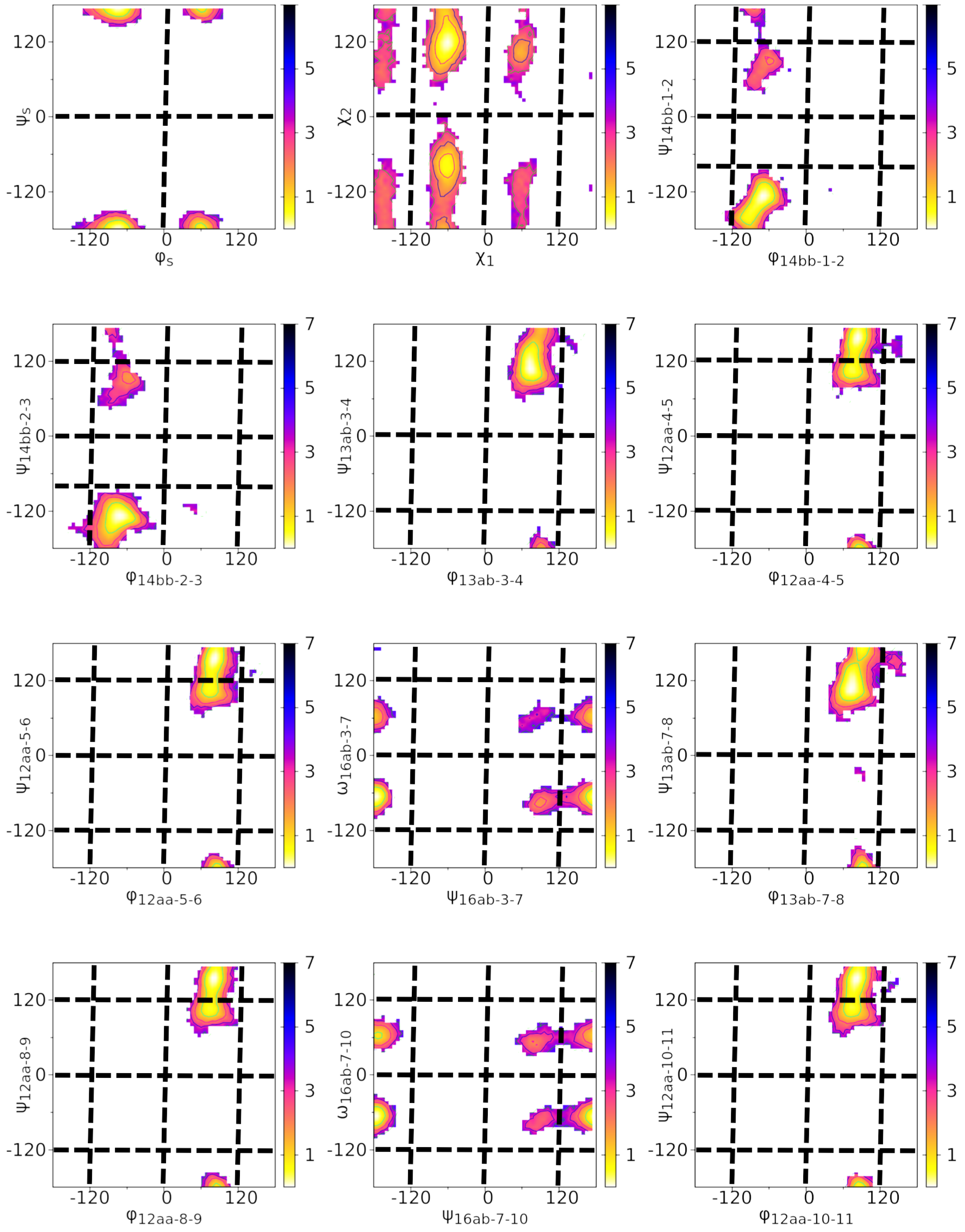

Figure S2b: Definitions of the integer values of the GL clusters for the M9 Glycan overlaid on 2D PMFs obtained from the simulation of M9 glycan with Asn dipeptide. The integer values are identical to those for the same type of glycosydic linkage of M5 glycan shown in Figure S2a.

Table S4. Definition of the dihedrals contributing to the 12 integer GL Cluster definitions for the M9 and M5 glycans. N indicates that the linkage is not present in the M5 glycan. See Figure S2 for the definitions of the GL cluster numbers for individual linkages and Figure S1 for the structural model of M5 and M9.

Number	M9 dihedrals	M5 dihedrals
1	φ_s/ψ_s	φ_s/ψ_s
2	protein $\chi 1/\chi 2$	protein $\chi 1/\chi 2$
	glycosidic linkages	glycosidic linkages
3	φ14-1-2/ψ14-1-2	φ14-1-2/ψ14-1-2
4	φ14-2-3/ψ14-2-3	φ14-2-3/ψ14-2-3
5	φ13-3-4/ψ13-3-4	φ13-3-4/ψ13-3-4
6	φ12-4-5/ψ12-4-5	N
7	φ12-5-6/ψ12-5-6	N
8	ψ16-3-7/ω16-3-7	ψ16-3-5/ω16-3-5
9	φ13-7-8/ψ13-7-8	φ13-5-7/ψ13-5-7
10	φ12-8-9/ψ12-8-9	N
11	ψ16-7-10/ω16-7-10	ψ16-5-6/ω16-5-7
12	φ12-10-11/ψ12-10-11	N

S.3 Free energy calculations

The free energy or potential of mean force (PMF) along one or two linkage dihedrals was computed from the unperturbed ground-state replica as,

$$\begin{aligned} G(\omega_i) &= \frac{-1}{\beta_0}\ln\left\{\int \rho(\mathbf{R})\,\delta\left(\Omega(\mathbf{R})-\omega_i\right)d\mathbf{R}\right\} \\ &= \frac{-1}{\beta_0}\ln\left\{\sum_{j=1}^{N_s}\Delta\left(\Omega(\mathbf{R}_i)-\omega_i\right)/N_s\right\} \end{aligned} \qquad \text{(S1)}$$

where N_s is the number of snapshots recorded in the ground-state replica and

$\Delta\left(\Omega\left(\mathbf{R}_j\right)-\omega_i\right)=1$ if $\Omega\left(\mathbf{R}_j\right)$ is within the bin $[w_i-\Delta w/2, w_i+\Delta w/2]$ and otherwise

$\Delta\left(\Omega\left(\mathbf{R}_j\right)-\omega_i\right)=0$. Only the recorded snapshots every 8 ps from the ground state replica were used in the free energy calculation here.

S.4 3D Cartesian coordinate volume description of glycan conformational sampling.

The spatial distribution or range of conformational space as defined by Cartesian coordinates can be represented by the distribution or volume of the sampled conformations. To compute the sampled volume, a 3D grid with a voxel size of 1Å×1Å×1Å was constructed around the saccharide molecule. Then, each snapshot in the ground-state replica trajectories from the HREST-BP simulation of the Env trimer model was aligned over the protein backbone non-hydrogen atoms. With such an alignment reference, the spatial distribution of saccharide relative to the protein surface is presented. For a given snapshot, each voxel was assigned a value of 1 if it was occupied by the pyranose ring or the covalently connected Asn non-hydrogen atoms.[22] The occupied volume is the total number of occupied voxels. This analysis was performed for all snapshots from the respective trajectories. Visualization of the sampled volume was performed by normalizing the voxel occupancies over all the snapshots. This approach allows for the volume sampled by selected portions of the saccharides as well as of the full saccharide to be quantified and visualized. To compare the similarity of the sampled conformational space between two glycans A and B the overlap coefficient (*OC*) was computed as follows,[23]

$$OC = \sum_{i=1}^{N} \min\left(\frac{n_i^A}{\sum_{j=1}^{N} n_j^A}, \frac{n_i^B}{\sum_{j=1}^{N} n_j^B} \right) \quad \text{(S2)}$$

where N is the number of voxels in the 3D grid map; n_i^A and n_i^B are the occupancies at the i-th voxel for glycans A and B, respectively.[23] From the definition of *OC*, *OC*=0 and 1 suggest the two glycans sampled completely different and identical regions in conformational space, respectively.

S.5 Reconstruction of antibody-Env trimer models

Antibody-Env trimer models were rebuilt from snapshots of the Env trimer simulation and selected crystal structures. A total of 375 frames, every 800 ps, were extracted from the ground-state replica of Env trimer trajectory. The antibodies were then built onto the trimer by overlaying the crystallographic gp120 protein non-hydrogen backbone atoms (or gp120 fragment) and extracting the respective antibody coordinates. Antibodies modeled onto the Env include PG9 (PDBID: 3U4E),[11] VRC01 (PDBID: 5FYJ),[24] PGT122 (PDBID: 4TVP and 5FYJ),[1,24] 35O22 (PDBID: 4TVP and 5FYJ),[1,24] 8ANC195 (PDBID: 5C7K),[25] PGT135 (PDBID: 4JM2),[8] and 17B (PDBID: 4JM2).[8] The 17B antibody clashes with portions of the Env protein due to the 4JM2 structure being obtained for HIV strain JR-FL though the resulting model was used for subsequent analysis. Based on the rebuilt models the 3D Cartesian coordinate volume distributions were calculated for the antibodies allowing the OC values with the individual glycans to be calculated. The complementarity determining regions (CDRs) of the antibodies were assigned in the corresponding PDB structures using Paratome [26,27] and OC values were also computed between each glycan and the CDRs.

S.6 Enhanced Conformational Sampling obtained with the HREST-BP methodology.

As the present study was designed to focus on the conformational heterogeneity of the glycans, only those structures of the simulation systems were subjected to enhanced sampling, with the details of that sampling described below. The protein portions of the systems were allowed to propagate with nearly unperturbed Hamiltonian during the HREST-BP simulations where only the interactions of the protein Asn side chains with the glycans were reformulated according to Eq. (1) in the main text. Root-mean square

difference (RMSD) analysis showed the protein structures to be stable, with RMSD values in the vicinity of 3.5 Å, 2.3 Å and 6.6 Å with respect to the crystal structure in HREST-BP simulation for the respective Env trimer, M9-gp120 core and M5-gp120 V1V2 loops. Similarly, for the standard MD simulation of Env an RMSD of 3.2 Å was obtained. A flip of a β sheet occurred in the simulation of the M5-gp120 V1V2 loops (Figure S3) resulting in the large RMSD value of 6.6 Å. In all assessed systems, the glycans showed substantial conformational diversity. The fully glycosylated monomer occluded the majority of the gp140 protein from the surrounding environment with the glycan shield extending out to 25 Å from the protomer, based on the Asn Nδ2 to glycan non-hydrogen atom maximum distances.

To effectively study glycan heterogeneity it is essential that the methodology samples a representative range of conformational space. Accordingly, initial analyses focused on the ability of the HREST-BP method to effectively sample the full range of glycan conformations as well as the ability to enhance sampling over MD alone. Shown in Figure S4 is the number of GL conformations sampled by each of the glycans in the ENV trimer system from the HREST-BP and standard MD simulations. As is evident, the number of GL conformations being sampled in the HREST-BP simulation is much larger than those seen in standard MD, demonstrating the utility of our enhanced sampling method. Accordingly, unless noted the analysis is based on the HREST-BP simulations.

The results shown in Figure S4 include the number of GL conformations sampled by the M5 and M9 glycans linked to a single Asn dipeptide as well as to the gp120 V1V2 loop for M5 and the gp120 core for the M9 glycan. When only the glycosidic linkages in

the M5 glycan are included in the analysis (ie. the $\chi1/\chi2$ link to the protein is not included), the number of GL cluster sampled in the Asn dipeptide and gp120 V1V2 loop systems are similar (118 vs 120), which is larger than that observed in all the M5 glycans from the Env trimer system (Figure S4a). However, when the $\chi1/\chi2$ dihedrals are included in the analysis (Figure S4b), the presence of the gp120 V1V2 loop limits the number of GL conformations accessible to the glycan to 140 compared to the 249 accessible conformations to the M5-Asn dipeptide (the number of GL clusters including $\chi1/\chi2$ and all glycosidic linkages) (Figure S4b). Interestingly, some of the M5 glycans in the Env sampled more than 140 individual GL clusters, indicating the impact of the local protein and glycans on conformational sampling by the M5 glycan. Similar results were seen for the M9 glycans (g332 and g392 in Figures 1), where there was a decrease in the number of GL clusters sampled upon going from the M9-Asn dipeptide system to the M9-gp120 core. However, all the M9 glycans in the Env trimer system sampled a smaller number of GL clusters than in the M9-gp120 core system. These results indicate that the extent of sampling of the glycans is typically decreased in the Env trimer compared to the much simpler model systems, indicating the importance of simulating the entire system. However, it is clear that the extent of sampling of the individuals glycans in the Env varies widely, indicating a significant impact of the local environment on the conformational sampling of each glycan.

Convergence of the HREST-BP simulations for all systems was checked based on the overlap of the Cartesian volumes sampled during the first and second half of the simulations. As may be seen in Figure S5, the overlap coefficients, OC, were typically in the vicinity of 0.7 to 0.8. As the OC values are non-linear, as previously discussed,[28]

these values represent a high level of similarity of the sampled regions of conformational space. Accordingly, while "absolute" convergence has not been achieved, the HREST-BP method yields a satisfactory level of convergence such that the studied ensemble of glycan conformations may be considered representative of the experimental regimen.

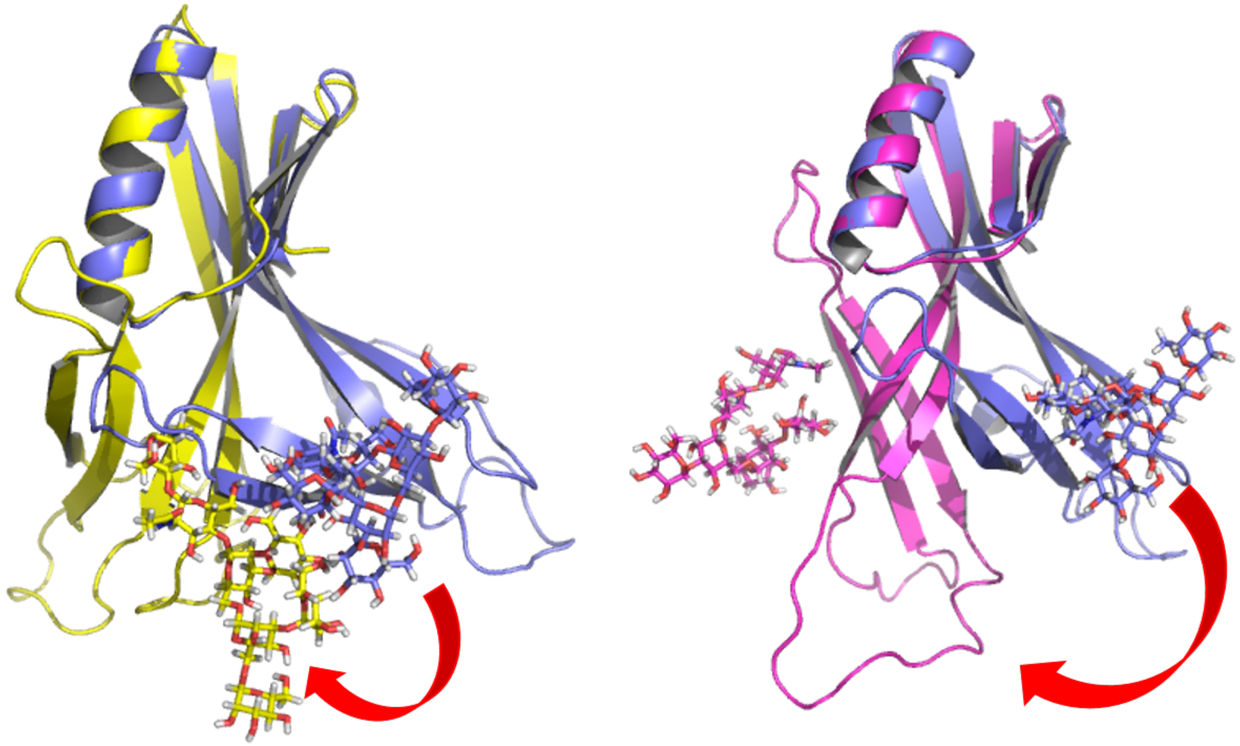

Figure S3. Flip of the β sheet in the HREST-BP simulation of the M5-gp120 V1V2 loops model. Two representative computational snapshots were shown in yellow and magenta. The crystal structure (PDBID: 4DQO) [7] is in blue. In the left panel, the glycans are on the same side of the β sheet; in right panel the glycans are on different sides of the β sheet, that is, a rotation around the sheet axis occurred in the two snapshots. Protein is shown in cartoon and glycans in stick model.

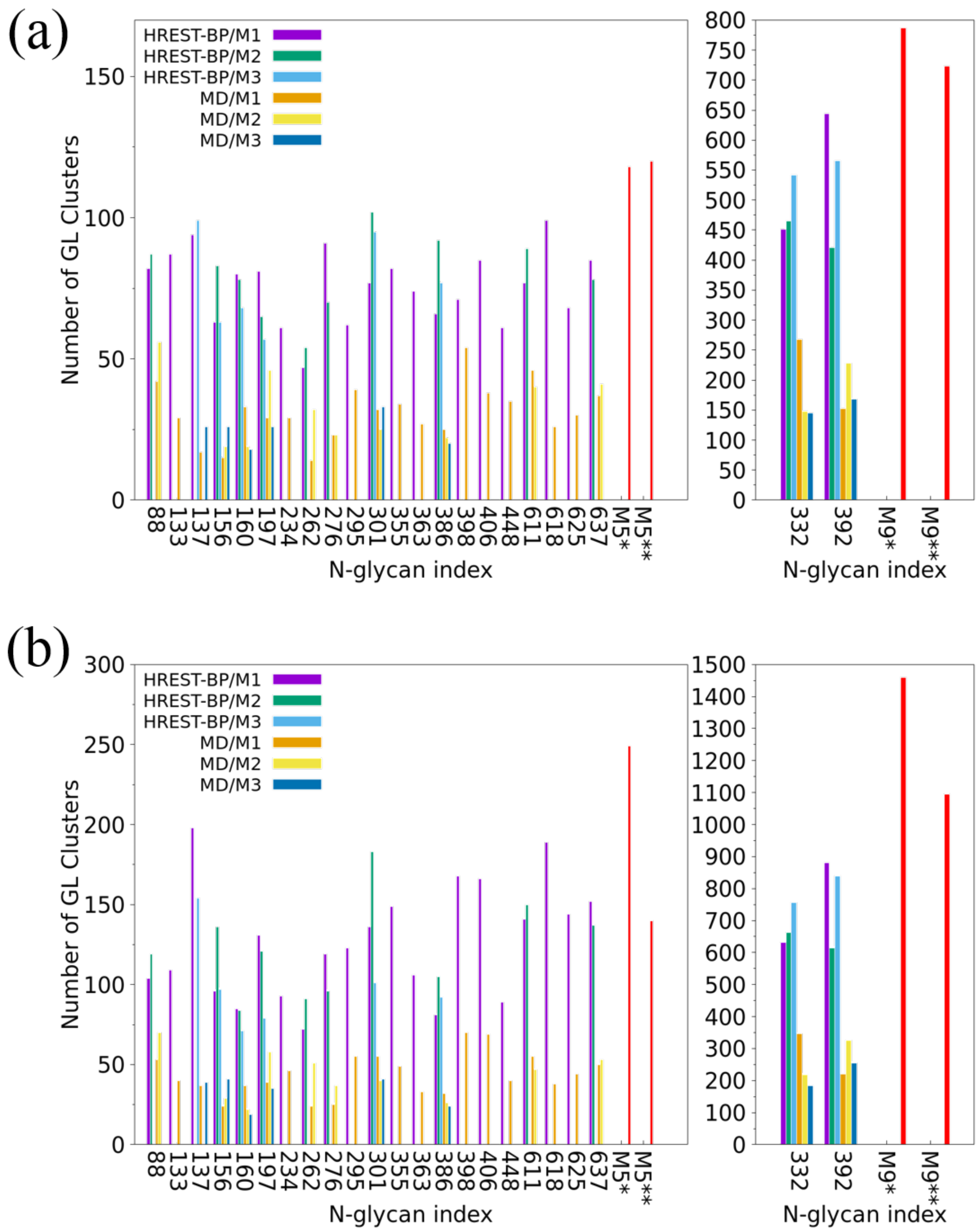
(a)
HREST-BP/M1
HREST-BP/M2
HREST-BP/M3
MD/M1
MD/M2
MD/M3
Number of GL Clusters
N-glycan index
88 133 137 156 160 197 234 262 276 295 301 355 363 386 398 406 448 611 618 625 637 M5* M5**
332 392 M9* M9**
(b)
HREST-BP/M1
HREST-BP/M2
HREST-BP/M3
MD/M1
MD/M2
MD/M3
Number of GL Clusters
N-glycan index
88 133 137 156 160 197 234 262 276 295 301 355 363 386 398 406 448 611 618 625 637 M5* M5**
332 392 M9* M9**

Figure S4. The number of sampled GL clusters of each N-glycan in the simulations of the Env with HREST-BP and standard MD. The M9 glycans are connected to Asn residues 332 and 392; and all other sites are glycosylated with M5 glycans. M1, M2 and M3 represent the three monomers in the Env model. The X-axis labels M5*, M5**, M9* and M9** are for the systems with M5 connected to an Asn dipeptide or the gp120 V1V2 loop and M9 connected to an Asn dipeptide or the gp120 core, respectively. Only HREST-BP simulations were performed for these systems. (A) Only the saccharide glycosidic linkage dihedrals or (B) all glycosidic linkage dihedrals, including $\chi1/\chi2$ in the link to Asn are included in the analyses.

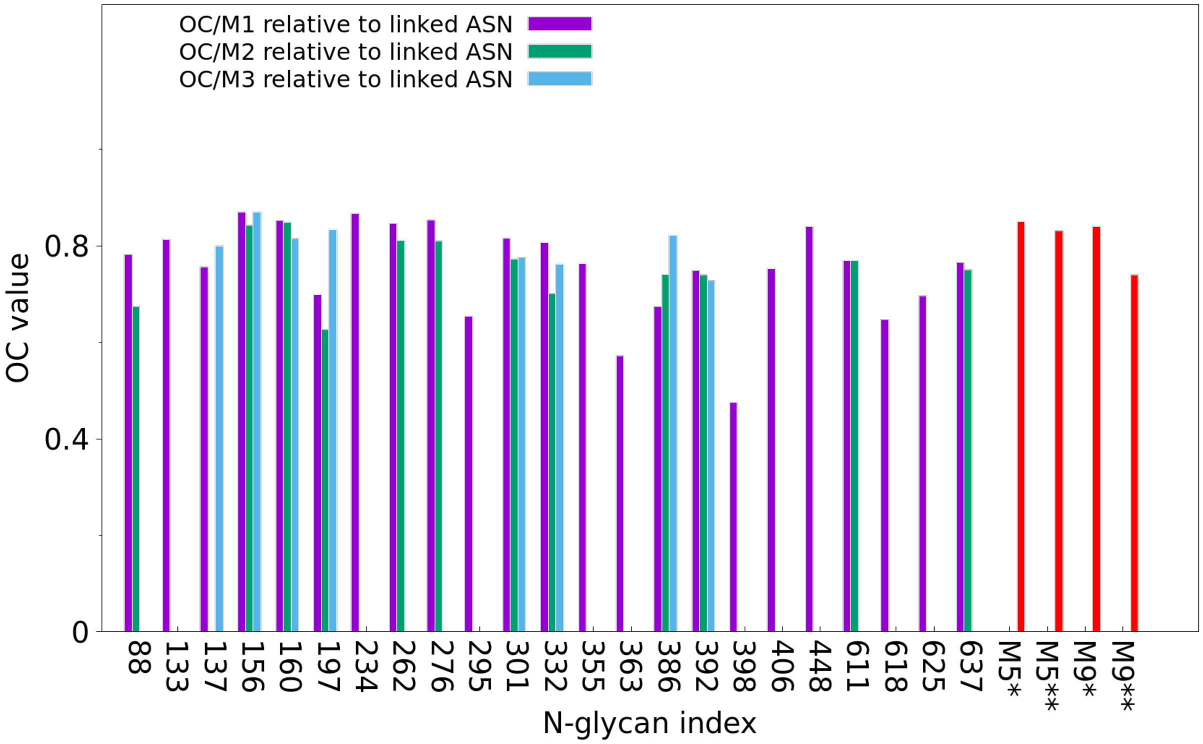

Figure S5. Convergence check with overlap coefficient (OC) of individual glycans computed between the first and second 150 ns halves of the full 300 ns trajectory from the HREST-BP simulation of the Env. The OC values were computed by aligning the heavy atoms of the linked Asn residue (OC/M1 relative to linked Asn, OC/M2 relative to linked Asn, OC/M3 relative to linked Asn). The X-axis labels M5*, M5**, M9* and M9** are for the systems with M5 connected to Asn dipeptide and the gp120 V1V2 loop models, M9 connected to Asn dipeptide and the gp120 core models, respectively. Only HREST-BP simulations were performed for these systems.

Table S5a. GL clusters sampled by glycan M1-g88 with a probability of 0.01 or more along with clusters from experimental structures from a survey of the PDB. See Figure S2 and Table S4 for the definitions of the GL cluster numbers for the individual linkages. Text in underlined bold in the Notes section indicates antibodies that bind to the analyzed glycan.

#	HREST2-BP 123456789012	Prob.	Experimental 123456789012	Notes on experimental structure (PDB ID)
1	11223NN63N6N	0.34	11223NN63N6N	4TVP: PGT122 and **35O22**, BG505.SOSIP.664[1]
2	11223NN63N7N	0.18	1122NNNNNNNN	5ACO: PGT128, BG505.SOSIP.664 cryoEM, R=4.4.[29]
3	11223NN63N4N	0.08	1122NNNNNNNN	"
4	11223NN73N6N	0.05	1122NNNNNNNN	"
5	11223NN33N6N	0.04	112NNNNNNNNN	5FUU: PGT151, JF-FL[30]
			112NNNNNNNNN	5FUU: PGT151, JF-FL Env trimer.[30]
6	10213NN63N7N	0.04	1122NNNNNNNN	"
7	31223NN63N7N	0.03	1022NNNNNNNN	"
8	31223NN63N6N	0.02	112238N85N7N	5FYJ: PGT122, **35O22**, VRC01, clade G X1193.c1.SOSIP.664.[24]
9	31223NN73N6N	0.02	11223NN45N6N	5FYK: PGT122, **35O22**, VRC34.01, BG505 SOSIP.664.[24]
10	30213NN63N7N	0.02		
11	11123NN63N7N	0.02		
12	11223NN73N7N	0.02	11225NN61NNN	"
13	10223NN63N7N	0.01	10223NN83N6N	5FYL: PGT122, **35O22**, Clade A BG505 SOSIP.664.[24]
14	10213NN63N4N	0.01		
15	11223NN63N3N	0.01		
16	31223NN63N4N	0.01		
17	11123NN63N4N	0.01		
18	11223NN73N4N	0.01		
19	11223NN33N7N	0.01		
20	31213NN63N7N	0.01		
21	10223NN73N6N	0.01		
22	11223NN43N6N	0.01		
23	30213NN63N4N	0.01		
Initial GL cluster			11223NN62N4N	

Table S5b. GL clusters sampled by glycan M1-g137 with a probability of 0.01 or more along with clusters from experimental structures from a survey of the PDB. See Figure S2 and Table S4 for the definitions of the GL cluster numbers for the individual linkages. Text in underlined bold in the Notes section indicates antibodies that bind to the analyzed glycan.

#	HREST2-BP 123456789012	Prob.	Experimental 123456789012	Notes on experimental structure (PDB ID)
1	31223NN63N7N	0.11		
2	11223NN63N6N	0.10		
3	34223NN63N7N	0.09		
4	14223NN63N7N	0.05		
5	14223NN63N6N	0.05	14283NNNNNNN	4TVP: **PGT122** and 35O22, BG505.SOSIP.664.[1]
6	31223NN63N6N	0.04		
7	35123NN63N7N	0.04		
8	11223NN73N6N	0.03		
9	34223NN63N4N	0.03		
10	34223NN73N6N	0.03		
11	31223NN63N4N	0.03		
12	10223NN63N6N	0.02		
13	11223NN63N7N	0.02		
14	35123NN63N4N	0.02		
15	34213NN63N7N	0.02		
16	14223NN73N6N	0.02	14253NN22N6N	5FYL: **PGT122**, 35O22, BG505 SOSIP.664.[24]
17	35223NN63N7N	0.02		
18	10223NN73N6N	0.02		
19	14213NN63N7N	0.01	14283NNNNNNN	5I8H: **PGT122**, VRC34.01, BG505 SOSIP.664.[31]
20	31223NN73N6N	0.01		
21	35213NN63N7N	0.01		
22	14223NN63N4N	0.01		
23	31123NN73N6N	0.01		
24	15223NN63N6N	0.01		
25	31223NN33N6N	0.01		
26	11223NN33N6N	0.01		
27	14213NN33N7N	0.01		
28	34223NN63N6N	0.01		
29	31223NN33N7N	0.01		
30	15223NN63N7N	0.01	15223NN63N7N	4NCO: **PGT122**, BG505 SOSIP.664.[9]
31	10223NN63N7N	0.01		
32	34223NN73N4N	0.01		
33	10123NN73N6N	0.01		
34	14213NN33N6N	0.01		
35	35213NN33N4N	0.01		
36	11223NN63N4N	0.01		
Initial GL cluster			14223NN63N7N	

Table S5c. GL clusters sampled by glycan M1-g156 with a probability of 0.01 or more along with clusters from experimental structures from a survey of the PDB. See Figure S2 and Table S4 for the definitions of the GL cluster numbers for the individual linkages. Text in underlined bold in the Notes section indicates antibodies that bind to the analyzed glycan.

	HREST-BP 123456789012	Prob.	Xtal 123456789012	Notes on experimental structure (PDB ID)
1	15223NN63N6N	0.50	15223NN73NNN	3U4E: **PG9** FAP, strain CAP45. [11]
2	15223NN63N7N	0.09	15223NNNNNNN	"
3	14223NN63N6N	0.08	14823NN63N7N	4NCO: **PGT122** Fab, BG505.SOSIP.[9]
4	15223NN73N6N	0.06	14823NN63N7N	"
5	15223NN33N6N	0.06	14823NN63N7N	"
6	15223NN73N7N	0.04	14223NN8NNNN	4TVP: **PGT122** and 35O22, BG505.SOSIP.664[1]
7	15223NN73N4N	0.03	1422NNNNNNNN	5ACO: **PGT128**, BG505.SOSIP.664 cryoEM.[29]
8	15223NN63N4N	0.02	1422NNNNNNNN	"
9	15223NN63N3N	0.02	1422NNNNNNNN	"
10	14223NN73N6N	0.01	142NNNNNNNNN	5C7K: **PGT128** and 8ANC195, BG505.SOSIP.[25]
11	14223NN63N7N	0.01	1522NNNNNNNN	5FUU: PGT151, JF-FL.[30]
12	14223NN33N6N	0.01	152NNNNNNNNN	"
13	14223NN73N7N	0.01	1522NNNNNNNN	"
			152734452205	5FYJ: **PGT122**, 35O22, VRC01, clade G X1193.c1.SOSIP.664.[24]
			142834585N6N	5FYK: **PGT122**, 35O22, VRC01, clade B JHR-FL SOSIP.664.[24]
			152235N63N45	5FYL: **PGT122**, 35O22, clade A BG505 SOSIP.664.[24]
			14223NN8NNNN	5I8H: **PGT122**, VRC34.01, BG505 SOSIP.664.[31]
			102232N43N8N	4DQO: **PG16**, strain ZM109, V1V2 region.[7]
Initial GL cluster			15223NN63N7N	

Table S5d. GL clusters sampled by glycan M1-g160 with a probability of 0.01 or more along with clusters from experimental structures from a survey of the PDB. See Figure S2 and Table S4 for the definitions of the GL cluster numbers for the individual linkages. Text in underlined bold in the Notes section indicates antibodies that bind to the analyzed glycan.

#	HREST2-BP 123456789012	Prob.	Experimental 123456789012	Notes on experimental structure (PDB ID)
1	11223NN63N6N	0.47		
2	11223NN63N7N	0.13		
3	11223NN73N6N	0.08	11223NN73N6N	3U2S: **PG9**.strain ZM109, V1V2 region;[11]
4	11223NN73N7N	0.08		
5	11223NN73N4N	0.05	11223NN73NNN	3U4E: **PG9.**strain CAP45, V1V2 region;[11]
6	11223NN63N4N	0.05		
7	11223NN33N6N	0.03		
8	11213NN63N6N	0.02	11223NN73N6N	4DQO: **PG16**, strain ZM109, V1V2 region.[7]
9	31223NN63N6N	0.02		
10	11223NN63N3N	0.01		
11	11223NN73N8N	0.01		
12	11213NN43N6N	0.01		
13	11223NN33N4N	0.01		
14	11223NN33N7N	0.01		
Initial GL cluster			11223NN73N6N	

Table S5e. GL clusters sampled by glycan M1-g276 with a probability of 0.01 or more along with clusters from experimental structures from a survey of the PDB. See Figure S2 and Table S4 for the definitions of the GL cluster numbers for the individual linkages. Text in underlined bold in the Notes section indicates antibodies that bind to the analyzed glycan.

	HREST-BP<br>123456789012	Prob.	Xtal<br>123456789012	Notes on experimental structure (PDB ID)
1	15223NN63N6N	0.33	15285NN7NN2N	4JKP: 45-46M2.[32]
21	15223NN63N6N	0.33	15285NN7NN2N	4JKP: 45-46M2.[32]2
2	15223NN63N7N	0.11	14223NN7NNNN	4P9H: **8ANC195** Fab, strain 93TH057.[33].2
	15223NN63N7N	0.11	14223NN7NNNN	4P9H: **8ANC195** Fab, strain 93TH057.[33].
3	15223NN73N6N	0.11	1528NNNNNNNN	4RQS: 17b Fab.[6]
4	15123NN63N7N	0.06	1522NNNNNNNN	5ACO: PGT128, BG505.SOSIP.664, cryoEM.[29]
5	35223NN63N6N	0.06	1522NNNNNNNN	“
6	15223NN63N4N	0.05	1522NNNNNNNN	“
7	15123NN63N4N	0.03	152234463N6N	5C7K: PGT128 and **8ANC195**,BG505.SOSIP. [25]6.
8	15223NN73N7N	0.03	142235N6NNNN	5CJX: **8ANC195**, BG505.SOSIP.[34].
9	35223NN63N7N	0.02	152235N6NNNN	“
10	15223NN33N6N	0.02	15223NN6NNNN	“
11	15223NN73N4N	0.02	1522NNNNNNNN	5FUU: PGT151, JF-FL Env trimer.[30]
12	15223NN33N7N	0.01	1522NNNNNNNN	“
13	15223NN33N4N	0.01	152NNNNNNNNN	“
14	15123NN63N6N	0.01	1527384837NN	5FYJ: PGT122, 35O22, **VRC01**<br>X1193.c1.SOSIP.664.[24]
15	15223NN63N3N	0.01	102728582NNN	5FYK: PGT122, 35O22, **VRC01**<br>JHR-FL SOSIP.664.[24]
16	11223NN63N6N	0.01	15212NN8NNNN	5FYL: PGT122, 35O22, BG505 SOSIP.664.[24]
17	35223NN73N6N	0.01	152235N635NN	5KZC: **VRC01**, M9 glycan at N276.[35]17
	35223NN73N6N	0.01	152235N635NN	5KZC: **VRC01**, M9 glycan at N276.[35]
18	14223NN63N6N	0.01	152235N635NN	“
19	35223NN63N4N	0.01	15223NN63NNN	“
20	15223NN43N6N	0.01		
Initial GL cluster			15223NN62N4N	

Table S5f. GL clusters sampled by glycan M1-g301 with a probability of 0.01 or more along with clusters from experimental structures from a survey of the PDB. See Figure S2 and Table S4 for the definitions of the GL cluster numbers for the individual linkages. Text in underlined bold in the Notes section indicates antibodies that bind to the analyzed glycan.

	HREST-BP 123456789012	Prob.	Xtal 123456789012	Notes on experimental structure (PDB ID)
1	14223NN63N6N	0.27	142NNNNNNNNN	4TVP: **PGT122**, 35O22, BG505.SOSIP.664.[1]
2	14223NN73N6N	0.20	142NNNNNNNNN	5FUU: PGT151, JF-FL Env trimer.[30]
3	15223NN73N6N	0.08	152234573N6N	5C7K: **PGT128**. 8ANC195, BG505.SOSIP.[25]
4	15223NN63N6N	0.06	15223NN73N6N	5ACO: **PGT128**, BG505.SOSIP.664, cryoEM.[29]
5	14223NN63N7N	0.06	14823NN63N7N	4NCO: **PGT122** Fab, BG505.SOSIP.[9]
6	14223NN33N6N	0.03		
7	14223NN63N4N	0.03	142NNNNNNNNN	5I8H: **PGT122**, VRC34.01,BG505 SOSIP.664.[31]
8	15223NN63N7N	0.02		
9	11223NN73N6N	0.02		
10	14223NN73N7N	0.02	14224NN2NN2N	5FYJ: **PGT122**, 35O22, VRC01, X1193.c1.SOSIP.664.[24]
11	14223NN43N6N	0.02	142235N243NN	5FYK: **PGT122**, 35O22, VRC01,JHR-FL SOSIP.664. [24]
12	14223NN73N4N	0.02		
13	15223NN73N7N	0.02	152724N22N5N	5FYL: **PGT122**, 35O22, BG505 SOSIP.664.[24]
14	15223NN63N4N	0.02		
15	35223NN73N4N	0.01		
16	14213NN33N6N	0.01		
17	35223NN73N7N	0.01		
18	15223NN73N4N	0.01		
19	15223NN33N6N	0.01		
20	14223NN43N7N	0.01		
21	14213NN63N6N	0.01		
22	14223NN63N3N	0.01		
Initial GL cluster			14223NN63N7N	

Table S5g. GL clusters sampled by glycan M1-g332 with a probability of 0.01 or more along with clusters from experimental structures from a survey of the PDB. See Figure S2 and Table S4 for the definitions of the GL cluster numbers for the individual linkages. Text in underlined bold in the Notes section indicates antibodies that bind to the analyzed glycan.

#	HREST-BP 123456789012	Prob.	Xtal 123456789012	Notes on experimental structure (PDB ID)
1	112235563565	0.11	112235N63N6N	4JM2: **<u>PGT135</u>** Fab, strain JR-FL.[8]
2	102235563565	0.05	1022345635NN	4NCO: **<u>PGT122</u>** Fab, BG505.SOSIP. [9]
3	112235563465	0.04	1022345635NN	"
4	112234563565	0.04	1022345635NN	"
5	112235563564	0.04	102234563N64	4R2G: **<u>PGT124</u>** Fab, strain JRCSF.[36]645
	112235563564	0.04	102234563N64	4R2G: **<u>PGT124</u>** Fab, strain JRCSF.[36]64
6	112235463565	0.03	112237563N64	"
7	102235563575	0.03	112234563N64	"
8	102235563564	0.02	112237563NNN	"
9	112234463565	0.02	112NNNNNNNNN	4RQS: 17b Fab.[6]
10	102234563565	0.02	11223546516N	4TVP: **<u>PGT122</u>**, 35O22, BG505.SOSIP.664.[1]
11	112235563464	0.02	102235563N65	5ACO: **<u>PGT128</u>**, BG505.SOSIP.664.[29]
12	112235573565	0.02	102235563N65	"
13	102235563475	0.02	102235563N65	"
14	102235563465	0.02	102235563N65	5C7K: **<u>PGT128</u>**, 8ANC195, BG505.SOSIP.[25]
15	112234563465	0.01	1022NNNNNNNN	5FUU: PGT151, JF-FL.[30]
16	102234563575	0.01	1022NNNNNNNN	"
17	102235463565	0.01	1122NNNNNNNN	"
18	112234563564	0.01	11223546356N	5FYJ: **<u>PGT122</u>**, 35O22, VRC01 X1193.c1.SOSIP.664. [24]
19	112235463465	0.01	10223546356N	5FYK: **<u>PGT122</u>**, 35O22, VRC01 JHR-FL SOSIP.664. [24]
20	112235463564	0.01	11223556356N	5FYL: **<u>PGT122</u>**, 35O22, BG505 SOSIP.664.[24]
21	102235463575	0.01	11223546516N	5I8H: **<u>PGT122</u>** VRC34.01, BG505 SOSIP.664.[31]
22	102235563574	0.01	11223546516N	"
23	312235563565	0.01		
24	102135563575	0.01		
25	102234563475	0.01		
26	102234463575	0.01		
27	112234573565	0.01		
28	112234463465	0.01		
29	102234463565	0.01		
30	102234563564	0.01		
31	112234463564	0.01		
32	112235563575	0.01		
33	112235573564	0.01		
34	102235563464	0.01		
35	112235533565	0.01		
36	102234563465	0.01		
37	102235463564	0.01		
38	312235463565	0.01		
39	112234563464	0.01		
40	102234463564	0.01		
41	112235463464	0.01		
Initial GL cluster			112234463164	

Table S5h. GL clusters sampled by glycan M1-g392 with a probability of 0.01 or more along with clusters from experimental structures from a survey of the PDB. See Figure S2 and Table S4 for the definitions of the GL cluster numbers for the individual linkages. Text in underlined bold in the Notes section indicates antibodies that bind to the analyzed glycan.

	HREST-BP 123456789012	Prob.	Xtal 123456789012	Notes on experimental structure (PDB ID)
1	142235573565	0.08	142234473N65	4JM2: **PGT135** Fab, strain JR-FL.[8]
2	142235573545	0.06	148NNNNNNNNN	4TVP: PGT122,35O22, BG505.SOSIP.664.[1]
3	142235573575	0.03	142NNNNNNNNN	5ACO: PGT128, BG505.SOSIP.664, cryoEM.[29]
4	142235573564	0.03	142NNNNNNNNN	"
5	142234573565	0.03	142NNNNNNNNN	"
6	142235473565	0.02	1422NNNNNNNN	5C7K: PGT128, 8ANC195, BG505.SOSIP.[25]
7	142235573465	0.02	1422NNNNNNNN	5FUU: PGT151, JF-FL.[30]
8	142234573545	0.02	142NNNNNNNNN	"
9	142235473545	0.02	1422NNNNNNNN	"
10	142234573575	0.02	14872NN83NNN	5FYK: PGT122, 35O22, VRC01, JHR-FL SOSIP.664.[24]
11	341235573565	0.02	148534N8NNNN	5FYL: PGT122, 35O22, BG505 SOSIP.664.[24]
12	142135533564	0.01	148NNNNNNNNN	5I8H: PGT122 VRC34.01, BG505 SOSIP.664.[31]
13	142235573445	0.01	1488NNNNNNNN	"
14	142235563565	0.01		
15	142234573564	0.01		
16	152235573565	0.01		
17	142235573574	0.01		
18	142234473565	0.01		
19	142235473575	0.01		
20	142235543465	0.01		
21	142235473564	0.01		
22	142235575575	0.01		
23	142235575475	0.01		
24	142234473545	0.01		
25	142235573464	0.01		
26	142134533564	0.01		
27	142234573574	0.01		
28	142234473575	0.01		
29	352235573565	0.01		
30	152234573565	0.01		
31	142234573465	0.01		
32	151235573575	0.01		
33	142235573475	0.01		
34	142235473465	0.01		
35	142235563465	0.01		
36	341235573564	0.01		
37	142235543464	0.01		
38	152235573564	0.01		
39	142235563564	0.01		
40	152235573465	0.01		
41	142234473564	0.01		
42	142234563565	0.01		
Initial GL cluster			142234473465	

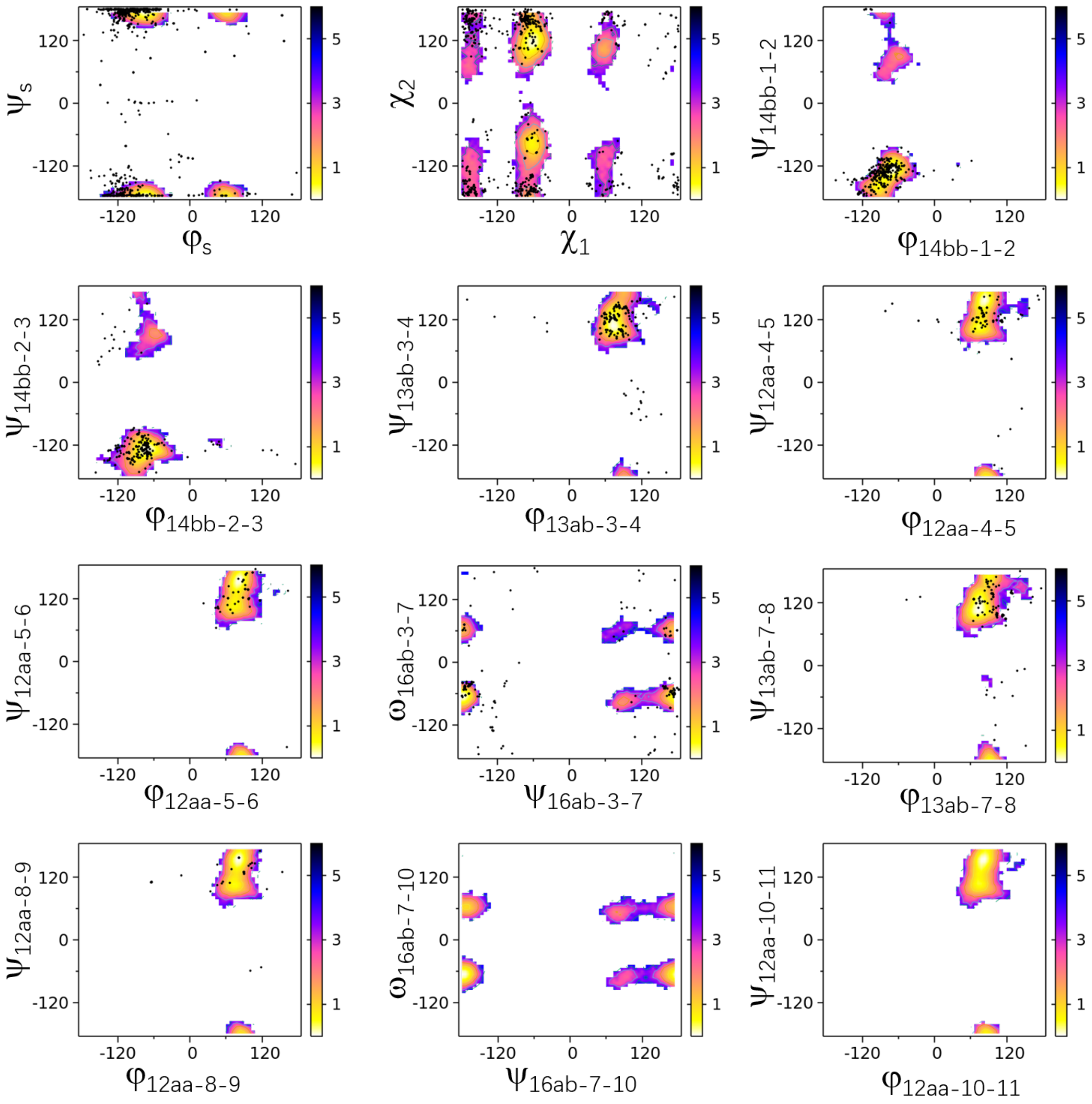

Figure S6. Potentials of mean forces for glycosidic linkages from the M9-ASN dipeptide HREST-BP enhanced sampling simulation and the distribution from glycosidic linkages from 298 crystal structures (black dots) selected from the protein data bank[37] with the search keyword "HIV gp120". Of the 298 PDBIDs, only 147 structures have glycosylated ASN residues.

Table S6. Glycan pairs in which finite overlap of the 3D Cartesian volumes was present from the HREST-BP simulation. # indicates the number of interacting pairs for the glycan and OC represent the overlap coefficient between the 3D volumes sampled by each glycan.

Monomer 1				Monomer 2				Monomer-3			
Glycan 1	#	Glycan 2	OC	Glycan 1	#	Glycan 2	OC	Glycan 1	#	Glycan 2	OC
M1-g88	6	M1-g234	0.0003	M2-g88	0			M3-g137	6	M3-g156	0.0227
		M1-g355	0.0070	M2-g156	4	M2-g160	0.0079			M3-g160	0.0001
		M1-g406	0.0007			M2-g301	0.0040			M3-g301	0.0365
		M1-g618	0.0306			M3-g160	0.0000			M3-g332	0.0111
		M1-g625	0.0756			M3-g197	0.0001			M3-g386	0.0007
		M2-g611	0.0012	M2-g160	4	M1-g156	0.0000			M3-g392	0.0014
M1-g133	8	M1-g137	0.0199			M1-g160	0.0095	M3-g156	5	M1-g160	0.0002
		M1-g156	0.0002			M2-g156	0.0079			M1-g197	0.0002
		M1-g160	0.0004			M3-g160	0.0063			M3-g137	0.0227
		M1-g197	0.0334	M2-g197	5	M1-g137	0.0000			M3-g160	0.0073
		M1-g332	0.0078			M1-g156	0.0000			M3-g301	0.0012
		M1-g363	0.0191			M1-g301	0.0018	M3-g160	5	M1-g160	0.0220
		M1-g386	0.0503			M2-g386	0.0049			M2-g156	0.0000
		M1-g392	0.0011			M2-g392	0.0000			M2-g160	0.0063
		M1-g137	0.0199	M2-g262	2	M2-g301	0.0087			M3-g137	0.0001
M1-g137	11	M1-g156	0.0341			M2-g332	0.0001			M3-g156	0.0073
		M1-g160	0.0009	M2-g276	2	M2-g392	0.0000	M3-g197	4	M2-g156	0.0001
		M1-g197	0.0000			M2-g637	0.0095			M2-g301	0.0047
		M1-g295	0.0014	M2-g301	4	M2-g156	0.0040			M3-g386	0.0297
		M1-g301	0.0841			M2-g262	0.0087			M3-g392	0.0000
		M1-g332	0.0683			M2-g332	0.0539	M3-g301	4	M3-g137	0.0365
		M1-g386	0.0001			M3-g197	0.0047			M3-g156	0.0012
		M1-g392	0.0025	M2-g332	4	M2-g262	0.0001			M3-g332	0.1490
		M1-g406	0.0012			M2-g301	0.0539			M3-g392	0.0000
		M2-g197	0.0000			M2-g386	0.0132	M3-g332	4	M3-g137	0.0111
M1-g156	6	M1-g133	0.0002			M2-g392	0.0441			M3-g301	0.1490
		M1-g137	0.0341	M2-g386	3	M2-g197	0.0049			M3-g386	0.0032
		M1-g160	0.0123			M2-g332	0.0132			M3-g392	0.0192
		M1-g301	0.0022			M2-g392	0.1226	M3-g386	4	M3-g137	0.0007
		M2-g160	0.0000	M2-g392	4	M2-g197	0.0000			M3-g197	0.0297
		M2-g197	0.0000			M2-g276	0.0000			M3-g332	0.0032
M1-g160	6	M1-g133	0.0004			M2-g332	0.0441			M3-g392	0.1236
		M1-g137	0.0009			M2-g386	0.1226	M3-g392	5	M3-g137	0.0014
		M1-g156	0.0123	M2-g611	2	M1-g88	0.0012			M3-g197	0.0000
		M2-g160	0.0095			M2-g637	0.0056			M3-g301	0.0000
		M3-g156	0.0002	M2-g637	2	M2-g276	0.0095			M3-g332	0.0192
		M3-g160	0.0220			M2-g611	0.0056			M3-g386	0.1236
M1-g197	5	M1-g133	0.0334								
		M1-g137	0.0000								
		M1-g363	0.0058								
		M1-g386	0.0079								
		M3-g156	0.0002								
M1-g234	6	M1-g88	0.0003								
		M1-g276	0.0771								
		M1-g355	0.0121								
		M1-g398	0.0000								
		M1-g625	0.0107								
		M1-g637	0.0466								

M1-g262	5	M1-g295	0.0176
		M1-g301	0.0113
		M1-g332	0.0000
		M1-g406	0.0001
		M1-g448	0.0114
M1-g276	5	M1-g234	0.0771
		M1-g355	0.0009
		M1-g398	0.0016
		M1-g625	0.0000
		M1-g637	0.0214
M1-g295	8	M1-g137	0.0014
		M1-g262	0.0176
		M1-g301	0.0073
		M1-g332	0.0372
		M1-g355	0.0000
		M1-g398	0.0000
		M1-g406	0.0488
		M1-g448	0.2043
M1-g301	6	M1-g137	0.0841
		M1-g156	0.0022
		M1-g262	0.0113
		M1-g295	0.0073
		M1-g332	0.0171
		M2-g197	0.0018
M1-g332	11	M1-g133	0.0078
		M1-g137	0.0683
		M1-g262	0.0000
		M1-g295	0.0372
		M1-g301	0.0171
		M1-g363	0.0019
		M1-g386	0.0263
		M1-g392	0.0542
		M1-g398	0.0000
		M1-g406	0.0273
		M1-g448	0.0000
M1-g355	9	M1-g88	0.0070
		M1-g234	0.0121
		M1-g276	0.0009
		M1-g295	0.0000
		M1-g392	0.0002
		M1-g398	0.0212
		M1-g406	0.1049
		M1-g448	0.0001
		M1-g625	0.0011
M1-g363	7	M1-g133	0.0191
		M1-g197	0.0058
		M1-g332	0.0019
		M1-g386	0.2211
		M1-g392	0.1942
		M1-g398	0.0215
		M1-g406	0.0005
M1-g386	8	M1-g133	0.0503
		M1-g137	0.0001
		M1-g197	0.0079
		M1-g332	0.0263
		M1-g363	0.2211

		M1-g392	0.0918
		M1-g398	0.0001
		M1-g406	0.0003
M1-g392	8	M1-g133	0.0011
		M1-g137	0.0025
		M1-g332	0.0542
		M1-g355	0.0002
		M1-g363	0.1942
		M1-g386	0.0918
		M1-g398	0.0999
		M1-g406	0.0471
M1-g398	10	M1-g234	0.0000
		M1-g276	0.0016
		M1-g295	0.0000
		M1-g332	0.0000
		M1-g355	0.0212
		M1-g363	0.0215
		M1-g386	0.0001
		M1-g392	0.0999
		M1-g406	0.0835
		M1-g448	0.0001
M1-g406	11	M1-g88	0.0007
		M1-g137	0.0012
		M1-g262	0.0001
		M1-g295	0.0488
		M1-g332	0.0273
		M1-g355	0.1049
		M1-g363	0.0005
		M1-g386	0.0003
		M1-g392	0.0471
		M1-g398	0.0835
		M1-g448	0.0561
M1-g611	2	M1-g618	0.0116
		M1-g637	0.0247
M1-g618	4	M1-g88	0.0306
		M1-g611	0.0116
		M1-g625	0.1278
		M1-g637	0.0013
M1-g625	6	M1-g88	0.0756
		M1-g234	0.0107
		M1-g276	0.0000
		M1-g355	0.0011
		M1-g618	0.1278
		M1-g637	0.0058
M1-g637	5	M1-g234	0.0466
		M1-g276	0.0214
		M1-g611	0.0247
		M1-g618	0.0013
		M1-g625	0.0058

Table S7a. Minimum distance analysis in Å of glycans with the antibodies modeled onto M1 of the HREST-BP simulated structures. The PDB identifiers of the structures used to model the antibodies are shown in parenthesis along with the number of glycans within 10 Å of the respective antibodies.[1,8,11,24,25] The largest minimum distances (termed Maximum) and the average minimum distance and the standard error (SE) are shown. Note that glycans with structural overlap with the antibodies show minimum distances less than 1 Å.

Glycan	Minimum	Maximum	Average	SE
PG9 (3U4E) 7 within 10 Å				
M1-g156	0.03	1.42	0.39	0.01
M2-g160	0.15	12.75	3.78	0.13
M1-g137	0.16	20.61	6.72	0.26
M1-g160	0.17	4.59	1.25	0.05
M1-g301	0.57	18.82	9.13	0.18
M3-g160	1.86	19.27	10.41	0.15
M2-g197	2.22	19.96	11.49	0.17
17b (4JM2) 14 within 10 Å				
M1-g156	0.04	0.84	0.37	0.01
M1-g160	0.07	0.81	0.39	0.01
M2-g160	0.08	4.87	0.77	0.03
M3-g160	0.10	4.79	0.62	0.03
M1-g133	0.11	5.90	1.99	0.06
M1-g197	0.20	4.66	1.82	0.06
M2-g197	0.21	7.85	2.73	0.09
M1-g137	0.23	18.76	8.72	0.28
M1-g301	0.63	15.74	7.46	0.16
M3-g156	0.83	17.84	9.61	0.18
M2-g156	1.02	16.56	8.23	0.16
M1-g386	1.39	11.16	8.08	0.06
M1-g363	3.24	18.15	12.52	0.15
M1-g332	8.00	21.77	16.93	0.13
CD4 receptor (4JM2) 13 within 10 Å				
M1-g197	0.10	10.16	1.95	0.11
M1-g363	0.33	11.87	6.34	0.13
M1-g276	0.52	12.20	5.43	0.12
M1-g386	1.05	15.29	9.61	0.13
M1-g637	1.12	29.94	12.24	0.32
M3-g301	1.43	34.97	16.73	0.37
M1-g392	1.43	20.26	15.04	0.15
M1-g234	1.63	17.61	9.63	0.14
M3-g137	1.99	45.12	24.64	0.45
M1-g133	3.44	23.80	13.31	0.18
M1-g398	4.15	31.09	17.65	0.29
M3-g156	5.68	29.57	15.62	0.21
M3-g332	9.63	41.44	26.80	0.38

PGT135 (4JM2) 12 within 10 Å				
M1-g392	0.04	6.47	0.59	0.04
M1-g133	0.06	8.54	2.67	0.11
M1-g332	0.07	5.21	1.07	0.06
M1-g386	0.10	4.74	1.00	0.04
M1-g137	0.13	18.23	6.48	0.28
M1-g406	0.18	23.60	9.97	0.34
M1-g363	0.20	9.68	3.89	0.12
M1-g398	0.25	22.66	10.50	0.30
M1-g301	3.11	19.22	14.69	0.10
M1-g295	4.82	13.48	10.00	0.10
M1-g197	4.90	24.07	12.02	0.16
M1-g160	9.61	25.99	23.04	0.11
35O22 (4TVP) 8 within 10 Å				
M1-g625	0.03	0.70	0.33	0.01
M1-g618	0.05	3.06	0.40	0.01
M1-g88	0.10	3.31	1.40	0.04
M1-g234	0.52	7.63	2.56	0.05
M1-g355	0.55	20.53	7.21	0.10
M1-g637	1.85	14.31	9.17	0.06
M1-g611	7.11	19.69	10.62	0.12
M1-g276	8.85	18.83	12.40	0.05
PGT122 (4TVP) 12 within 10 Å				
M1-g332	0.03	2.26	0.37	0.01
M1-g137	0.06	2.37	0.54	0.02
M1-g301	0.08	7.24	2.44	0.08
M1-g156	0.47	9.03	5.71	0.08
M1-g133	0.84	17.37	11.60	0.17
M1-g392	1.96	18.17	14.34	0.12
M1-g406	2.27	35.67	33.13	0.20
M1-g386	2.65	9.89	5.83	0.07
M1-g160	7.10	25.55	21.81	0.13
M1-g363	7.23	17.88	12.61	0.14
M1-g295	8.36	13.14	9.54	0.04
M1-g262	8.49	18.47	10.41	0.07
35O22 (5FYJ) 10 within 10 Å				
M1-g625	0.05	0.67	0.34	0.01
M1-g618	0.06	3.71	0.61	0.02
M1-g88	0.09	2.93	0.80	0.03
M1-g234	0.10	9.34	3.67	0.10
M1-g637	0.23	18.69	8.58	0.22
M1-g355	0.55	23.07	9.84	0.28
M1-g276	6.14	19.26	13.38	0.12
M1-g406	6.17	45.66	27.86	0.44
M1-g611	6.71	21.27	15.08	0.16
M1-g398	9.52	36.62	27.84	0.28

PGT122 (5FYJ) 12 within 10 Å				
M1-g137	0.04	5.17	0.59	0.03
M1-g332	0.06	4.95	0.74	0.04
M1-g301	0.14	8.52	3.62	0.09
M1-g386	0.16	10.76	7.51	0.12
M1-g156	0.41	9.98	4.89	0.09
M1-g133	0.84	16.41	9.57	0.21
M1-g392	1.64	17.36	9.81	0.17
M1-g295	1.71	14.18	10.20	0.15
M1-g406	2.19	36.42	23.81	0.39
M1-g363	6.86	16.86	12.73	0.08
M1-g262	7.04	18.54	12.69	0.14
M1-g160	7.10	24.68	20.46	0.13
VRC01 (5FYJ) 14 within 10 Å				
M1-g276	0.04	1.48	0.43	0.01
M1-g197	0.12	7.05	1.79	0.09
M1-g234	0.13	8.99	2.28	0.10
M1-g363	0.16	9.44	3.95	0.13
M1-g386	0.41	16.71	8.52	0.18
M1-g637	0.53	25.39	6.89	0.26
M1-g392	1.12	18.42	12.68	0.14
M1-g398	1.85	23.88	12.47	0.24
M1-g133	5.91	24.26	13.22	0.16
M1-g355	6.11	20.75	15.05	0.15
M3-g301	7.42	32.92	20.43	0.26
M1-g625	7.71	33.77	25.32	0.35
M3-g156	7.88	30.07	19.36	0.22
M3-g332	9.73	39.44	28.11	0.26
8ANC195 (5C7K) 9 within 10 Å				
M1-g637	0.03	4.66	0.64	0.04
M1-g276	0.05	3.27	0.71	0.03
M1-g234	0.05	0.77	0.35	0.01
M1-g611	0.06	11.73	5.27	0.18
M1-g625	0.11	14.83	7.16	0.24
M1-g618	0.16	14.13	5.44	0.23
M1-g355	0.52	15.04	8.54	0.16
M1-g88	1.31	14.37	10.20	0.12
M1-g398	4.99	25.49	16.01	0.21

Table 7b. Minimum distance analysis in Å of glycans with the CDR regions of the antibodies modeled onto M1 of the HREST-BP simulated structures. The PDB identifiers of the structures used to model the antibodies are shown in parenthesis along with the number of glycans within 10 Å of the respective antibodies.[1,8,11,24,25] The largest minimum distances (termed Maximum) and the average minimum distance and the standard error (SE) are shown. Note that glycans with structural overlap with the antibodies show minimum distances less than 1 Å

Glycan	Minimum	Maximum	Average	SE
PG9 (3U4E) 7 within 10 Å				
M1-g156	0.03	1.42	0.40	0.01
M2-g160	0.15	12.75	3.78	0.13
M1-g137	0.16	24.31	9.36	0.33
M1-g160	0.17	4.59	1.25	0.05
M3-g160	1.86	19.27	10.41	0.15
M2-g197	2.22	19.96	11.73	0.17
M1-g301	2.78	19.62	10.63	0.15
17b (4JM2) 13 within 10 Å				
M1-g156	0.04	1.09	0.51	0.01
M1-g133	0.11	7.48	2.60	0.09
M1-g160	0.25	2.43	1.32	0.03
M2-g197	0.29	7.85	3.33	0.09
M1-g197	0.30	4.66	2.08	0.05
M1-g137	0.42	18.76	9.28	0.25
M1-g386	1.39	11.16	8.08	0.06
M2-g160	2.47	17.50	10.18	0.13
M1-g363	3.24	18.15	12.52	0.15
M1-g301	3.24	15.74	8.74	0.12
M3-g160	3.55	17.75	11.04	0.13
M3-g156	7.99	26.46	18.02	0.21
M1-g332	8.00	21.77	16.93	0.13
CD4 receptor (4JM2)				
Not applicable				
PGT135 (4JM2) 11 within 10 Å				
M1-g133	0.06	9.85	3.18	0.11
M1-g392	0.06	7.16	0.70	0.05
M1-g332	0.07	5.24	1.11	0.06
M1-g386	0.10	4.74	1.09	0.04
M1-g137	0.13	18.23	7.20	0.27
M1-g406	0.18	23.60	10.25	0.34
M1-g398	0.25	23.71	11.68	0.31
M1-g363	0.26	9.68	4.79	0.10
M1-g301	3.11	19.22	14.69	0.10
M1-g295	4.82	13.48	10.00	0.10
M1-g197	4.90	24.07	12.03	0.16

35O22 (4TVP) 7 within 10 Å				
M1-g625	0.03	0.85	0.36	0.01
M1-g618	0.05	3.06	0.45	0.01
M1-g88	0.10	3.31	1.40	0.04
M1-g234	1.61	16.21	9.50	0.07
M1-g355	5.69	28.02	16.45	0.12
M1-g611	8.19	19.69	10.94	0.12
M1-g637	8.37	19.63	15.75	0.07
PGT122 (4TVP) 11 within 10 Å				
M1-g332	0.03	2.26	0.37	0.01
M1-g137	0.06	3.93	0.91	0.04
M1-g156	0.91	9.03	5.72	0.07
M1-g392	1.96	18.17	14.34	0.12
M1-g406	2.27	35.67	33.13	0.20
M1-g301	2.37	11.49	6.87	0.09
M1-g386	2.65	9.89	5.83	0.07
M1-g133	3.49	19.42	14.25	0.15
M1-g160	7.10	25.55	21.85	0.12
M1-g363	7.23	17.88	12.61	0.14
M1-g295	8.36	13.14	9.54	0.04
35O22 (5FYJ) 7 within 10 Å				
M1-g625	0.05	1.73	0.39	0.01
M1-g88	0.09	2.93	0.81	0.03
M1-g618	0.10	4.89	0.95	0.04
M1-g234	1.25	17.69	10.78	0.16
M1-g637	1.90	26.27	14.57	0.23
M1-g355	5.11	31.16	17.54	0.28
M1-g611	8.27	21.27	15.66	0.14
PGT122 (5FYJ) 11 within 10 Å				
M1-g137	0.04	8.14	0.75	0.05
M1-g332	0.06	4.95	0.78	0.04
M1-g386	0.16	10.76	7.51	0.12
M1-g301	0.41	11.94	7.85	0.12
M1-g156	0.91	9.98	5.02	0.08
M1-g133	1.13	16.51	10.79	0.19
M1-g392	1.64	17.36	9.81	0.17
M1-g295	1.71	14.18	10.37	0.14
M1-g406	2.19	36.42	23.81	0.39
M1-g363	6.86	16.86	12.73	0.08
M1-g160	7.10	24.68	20.53	0.13
VRC01 (5FYJ) 11 within 10 Å				
M1-g276	0.05	2.33	0.57	0.02
M1-g197	0.12	15.58	6.27	0.21
M1-g234	0.13	10.01	2.58	0.11
M1-g637	0.53	25.39	7.95	0.26

M1-g363	1.92	13.97	9.35	0.10
M1-g386	2.89	17.03	11.98	0.10
M1-g355	6.11	21.41	15.13	0.15
M1-g398	6.25	30.24	17.57	0.25
M1-g392	7.11	22.49	17.14	0.12
M1-g133	9.83	29.74	19.14	0.19
M1-g625	9.85	37.40	28.80	0.40
8ANC195 (5C7K) 8 within 10 Å				
M1-g637	0.03	4.66	0.70	0.04
M1-g234	0.06	1.15	0.46	0.01
M1-g625	0.11	14.83	7.56	0.23
M1-g276	0.21	6.15	1.97	0.06
M1-g618	0.21	14.30	5.94	0.22
M1-g355	0.52	22.29	12.74	0.23
M1-g611	0.73	12.53	7.80	0.14
M1-g88	1.31	14.37	10.20	0.12

Table S8. Overlap coefficients between the rebuilt antibodies or the CD4 receptor[1,8,11,24,25] and individual glycans for pairs with finite OC values. Values of 0.0000 indicate overlap is occurring beyond the presented significant figures.

Glycan	PG9[a]	Glycan	PG9[b]	Glycan	PG9[c] (3U4E)
M1-g137	0.0082	M1-g160	0.0001	M1-g133	0.0000
M1-g156	0.0568	M2-g156	0.0493	M1-g160	0.0065
M1-g160	0.0139	M2-g160	0.0119	M1-g197	0.0005
M1-g301	0.0001	M2-g301	0.0000	M2-g160	0.0001
M2-g160	0.0060	M3-g160	0.0035	M3-g137	0.0171
M2-g197	0.0000	M3-g197	0.0000	M3-g156	0.0573
M3-g160	0.0000			M3-g160	0.0159
Glycan	17B[a]	Glycan	17B[b]	Glycan	17Bc (4JM2)
M1-g133	0.0061	M1-g160	0.0559	M1-g133	0.0001
M1-g137	0.0084	M2-g156	0.0650	M1-g137	0.0016
M1-g156	0.0712	M2-g160	0.0555	M1-g156	0.0023
M1-g160	0.0718	M2-g197	0.0038	M1-g160	0.0604
M1-g197	0.0080	M2-g301	0.0016	M1-g197	0.0078
M1-g301	0.0004	M3-g137	0.0033	M2-g156	0.0002
M1-g386	0.0000	M3-g156	0.0005	M2-g160	0.0446
M2-g156	0.0001	M3-g160	0.0374	M3-g137	0.0017
M2-g160	0.0356	M3-g197	0.0028	M3-g156	0.0726
M2-g197	0.0056			M3-g160	0.0635
M3-g156	0.0000			M3-g197	0.0039
M3-g160	0.0418			M3-g386	0.0000
Glycan	CD4[a]	Glycan	CD4[b]	Glycan	CD4c (4JM2)
M1-g133	0.0000	M1-g137	0.0001	M1-g276	0.0000
M1-g197	0.0398	M1-g276	0.0000	M2-g301	0.0336
M1-g234	0.0000	M1-g301	0.0119	M3-g197	0.0283
M1-g276	0.0000	M2-g197	0.0401	M3-g386	0.0006
M1-g363	0.0083	M2-g276	0.0011	M3-g392	0.0001
M1-g386	0.0018	M2-g386	0.0041		
M1-g392	0.0002	M2-g392	0.0005		
M1-g398	0.0000	M2-g637	0.0000		
M1-g637	0.0002				
M3-g137	0.0000				
M3-g301	0.0000				
Glycan	PGT135[a]	Glycan	PGT135[b]	Glycan	PGT135c (4JM2)
M1-g133	0.0220	M2-g301	0.0000	M3-g137	0.0316
M1-g137	0.0286	M2-g332	0.0821	M3-g197	0.0000
M1-g295	0.0000	M2-g386	0.0078	M3-g301	0.0009
M1-g301	0.0000	M2-g392	0.0694	M3-g332	0.0700
M1-g332	0.0758			M3-g386	0.0398
M1-g363	0.0071				
M1-g386	0.0275				
M1-g392	0.0844				
M1-g398	0.0042				
M1-g406	0.0284				
Glycan	35O22[a]	Glycan	35O22[b]	Glycan	35O22c (4TVP)

M1-g88	0.0489	M2-g88	0.0462		£™£
M1-g234	0.0078	M2-g637	0.0011		
M1-g355	0.0005				
M1-g618	0.1071				
M1-g625	0.1951				
M1-g637	0.0013				

Glycan	PGT122[a]	Glycan	PGT122[b]	Glycan	PGT122c (4TVP)
M1-g133	0.0007	M2-g156	0.0006	M3-g137	0.1542
M1-g137	0.1290	M2-g301	0.0142	M3-g156	0.0024
M1-g156	0.0007	M2-g332	0.0467	M3-g301	0.0800
M1-g295	0.0003	M2-g386	0.0000	M3-g332	0.0591
M1-g301	0.0102	M2-g392	0.0001	M3-g386	0.0005
M1-g332	0.0431			M3-g392	0.0014
M1-g386	0.0003				
M1-g392	0.0000				
M1-g406	0.0000				

Glycan	35O22[a]	Glycan	35O22[b]	Glycan	35O22c (5FYJ)
M1-g88	0.0489	M2-g88	0.0462		
M1-g234	0.0078	M2-g637	0.0011		
M1-g355	0.0005				
M1-g618	0.1071				
M1-g625	0.1951				
M1-g637	0.0013				

Glycan	PGT122[a]	Glycan	PGT122[b]	Glycan	PGT122c (5FYJ)
M1-g133	0.0007	M2-g156	0.0006	M2-g611	0.0000
M1-g137	0.1290	M2-g301	0.0142	M3-g137	0.1542
M1-g156	0.0007	M2-g332	0.0467	M3-g156	0.0024
M1-g295	0.0003	M2-g386	0.0000	M3-g301	0.0800
M1-g301	0.0102	M2-g392	0.0001	M3-g332	0.0591
M1-g332	0.0431	M2-g611	0.0000	M3-g386	0.0005
M1-g386	0.0003			M3-g392	0.0014
M1-g392	0.0000				
M1-g406	0.0000				
M2-g611	0.0000				

Glycan	VRC01[a]	Glycan	VRC01[b]	Glycan	VRC01c (5FYJ)
M1-g197	0.0424	M1-g262	0.0005	M2-g262	0.0023
M1-g234	0.0099	M1-g301	0.0076	M2-g301	0.0303
M1-g276	0.1459	M2-g197	0.0459	M2-g611	0.0000
M1-g355	0.0001	M2-g276	0.1015	M3-g197	0.0286
M1-g363	0.0219	M2-g386	0.0120	M3-g386	0.0018
M1-g386	0.0032	M2-g392	0.0014	M3-g392	0.0007
M1-g392	0.0007	M2-g611	0.0000		
M1-g398	0.0004	M2-g637	0.0001		
M1-g637	0.0015				
M2-g611	0.0000				

Glycan	8ANC195[a]	Glycan	8ANC195[b]	Glycan	8ANC195c (5C7K)
M1-g88	0.0002	M2-g276	0.0505		
M1-g234	0.1296	M2-g611	0.0088		
M1-g276	0.0934	M2-g637	0.0695		
M1-g355	0.0034				

M1-g398	0.0000
M1-g611	0.0171
M1-g618	0.0309
M1-g625	0.0222
M1-g637	0.1619

a. Antibody rebuilt on monomer 1.
b. Antibody rebuilt on monomer 2.
c. Antibody rebuilt on monomer 3.

Table S9. Overlap coefficient between CDRs of the rebuilt antibody[1,8,11,24,25] and individual glycans for pairs with finite OC values. Values of 0.0000 indicate overlap is occurring beyond the presented significant figures.

Glycan	PG9[a]	Glycan	PG9[a]	Glycan	PG9[c] (3U4E)
M1-g137	0.0116	M1-g160	0.0003	M1-g133	0.0000
M1-g156	0.1880	M2-g156	0.1640	M1-g160	0.0184
M1-g160	0.0428	M2-g160	0.0337	M1-g197	0.0015
M1-g301	0.0000	M2-g301	0.0001	M2-g160	0.0003
M2-g160	0.0172	M3-g160	0.0088	M3-g137	0.0101
M2-g197	0.0000	M3-g197	0.0001	M3-g156	0.1968
M3-g160	0.0001			M3-g160	0.0472
ycan	17B[a]	Glycan	17B[a]	Glycan	17Bc (4JM2)
M1-g133	0.0142	M2-g156	0.0843	M1-g197	0.0048
M1-g137	0.0061	M2-g160	0.0111	M3-g137	0.0006
M1-g156	0.0877	M2-g197	0.0092	M3-g156	0.0901
M1-g160	0.0179	M2-g301	0.0001	M3-g160	0.0188
M1-g197	0.0129	M3-g197	0.0035	M3-g197	0.0122
M1-g301	0.0000				
M1-g386	0.0000				
M2-g160	0.0000				
M2-g197	0.0066				
Glycan	PGT135[a]	Glycan	PGT135[a]	Glycan	PGT135c (4JM2)
M1-g133	0.0468	M2-g301	0.0000	M3-g137	0.0070
M1-g137	0.0401	M2-g332	0.2400	M3-g197	0.0001
M1-g295	0.0000	M2-g386	0.0170	M3-g301	0.0011
M1-g301	0.0001	M2-g392	0.1283	M3-g332	0.1076
M1-g332	0.2229				
M1-g363	0.0117				
M1-g386	0.0829				
M1-g392	0.2007				
M1-g398	0.0062				
M1-g406	0.0511				
Glycan	35O22[a]	Glycan	35O22[a]	Glycan	35O22c (4TVP)
M1-g88	0.0879	M2-g88	0.1285		
M1-g234	0.0001				
M1-g618	0.1211				
M1-g625	0.3206				
M1-g637	0.0000				

Glycan	PGT122[a]	Glycan	PGT122[a]	Glycan	PGT122c (4TVP)
M1-g133	0.0015	M2-g156	0.0001	M3-g137	0.1528
M1-g137	0.2674	M2-g301	0.0153	M3-g156	0.0014
M1-g156	0.0004	M2-g332	0.1341	M3-301	0.1254
M1-g295	0.0011	M2-g386	0.0000	M3-g332	0.1229
M1-g301	0.0074	M2-g392	0.0003	M3-g386	0.0011
M1-g332	0.1276			M3-g392	0.0029
M1-g386	0.0010				
M1-g392	0.0001				
M1-g406	0.0001				
Glycan	35O22[a]	Glycan	35O22[a]	Glycan	35O22[c] (5FYJ)
M1-g88	0.0879	M2-g88	0.1285		
M1-g234	0.0001				
M1-g618	0.1211				
M1-g625	0.3206				
M1-g637	0.0000				
Glycan	PGT122[a]	Glycan	PGT122[a]	Glycan	PGT122c (5FYJ)
M1-g133	0.0015	M2-g156	0.0001	M3-g137	0.1528
M1-g137	0.2674	M2-g301	0.0153	M3-g156	0.0014
M1-g156	0.0004	M2-g332	0.1341		
M1-g295	0.0011	M2-g386	0.0000		
M1-g301	0.0074	M2-g392	0.0003		
M1-g332	0.1276				
M1-g386	0.0010				
M1-g392	0.0001				
M1-g406	0.0001				
Glycan	VRC01[a]	Glycan	VRC01[a]	Glycan	VRC01c (5FYJ)
M1-g197	0.0159	M2-g197	0.0218	M2-g301	0.0003
M1-g234	0.0175	M2-g276	0.1023	M3-g197	0.0083
M1-g276	0.1459	M2-g386	0.0002		
M1-g355	0.0001	M2-g637	0.0001		
M1-g363	0.0007				
M1-g386	0.0002				
M1-g637	0.0012				
Glycan	8ANC195[a]	Glycan	8ANC195[b]	Glycan	8ANC195c (5C7K)
M1-g88	0.0003	M2-g276	0.0101		
M1-g234	0.1456	M2-g611	0.0010		
M1-g276	0.0200	M2-g637	0.1999		
M1-g355	0.0010				
M1-g611	0.0010				
M1-g618	0.0140				
M1-g625	0.0287				
M1-g637	0.2449				

a. Antibody rebuilt on monomer 1.
b. Antibody rebuilt on monomer 2.
c. Antibody rebuilt on monomer 3.

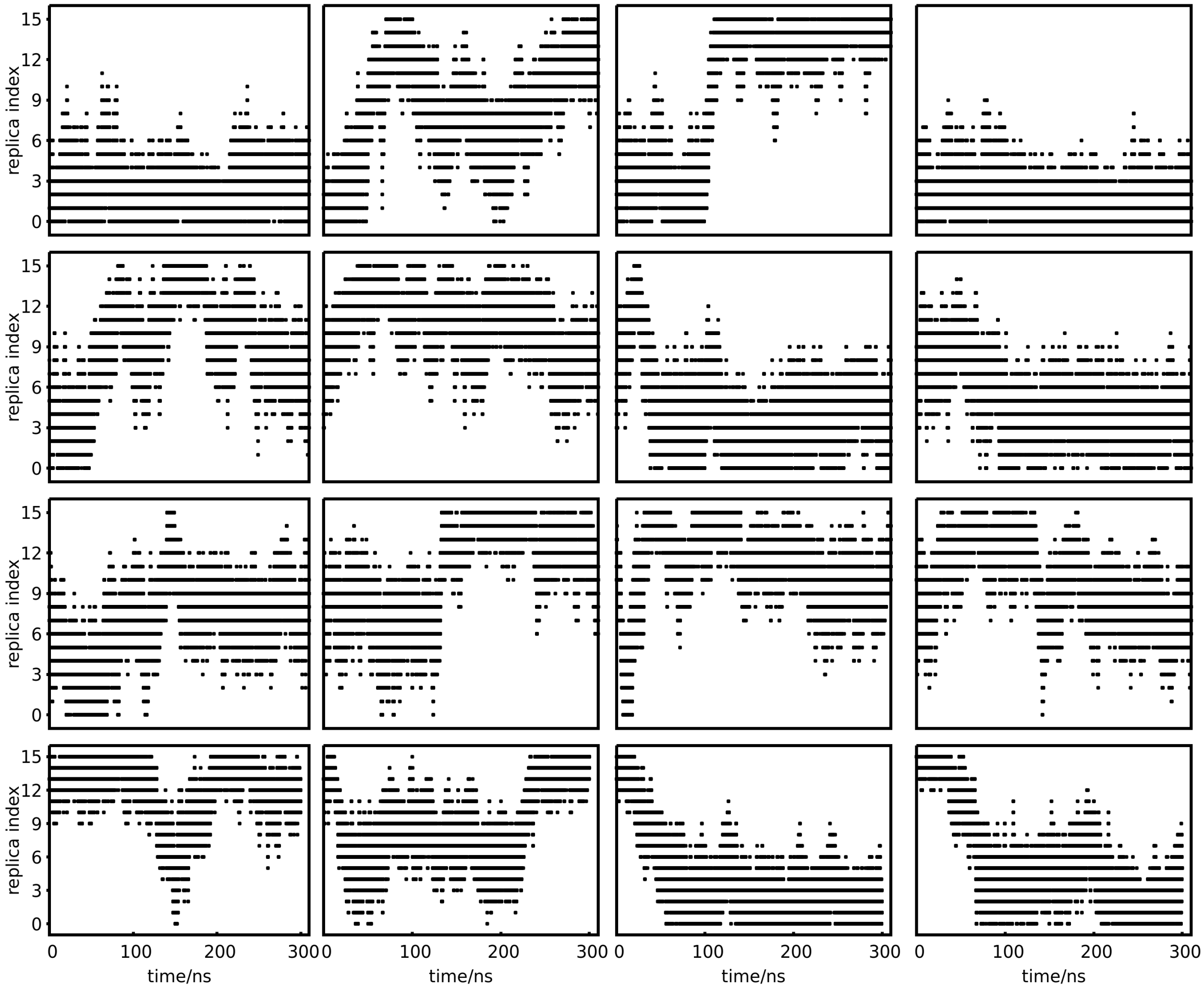

Figure S7. Random walk of the individual replica in the space of effective temperature and scaling factor λ. The top row shows replicas 0-3 from left to right, second row for replicas 4-7, third row for replicas 8-11 and 4th row for replicas 12-15. Analysis of the random walks shows the higher replicas to sample the ground state (replica 0) during the 300 ns HREST-BP simulation, indicating that efficient sampling was occurring.

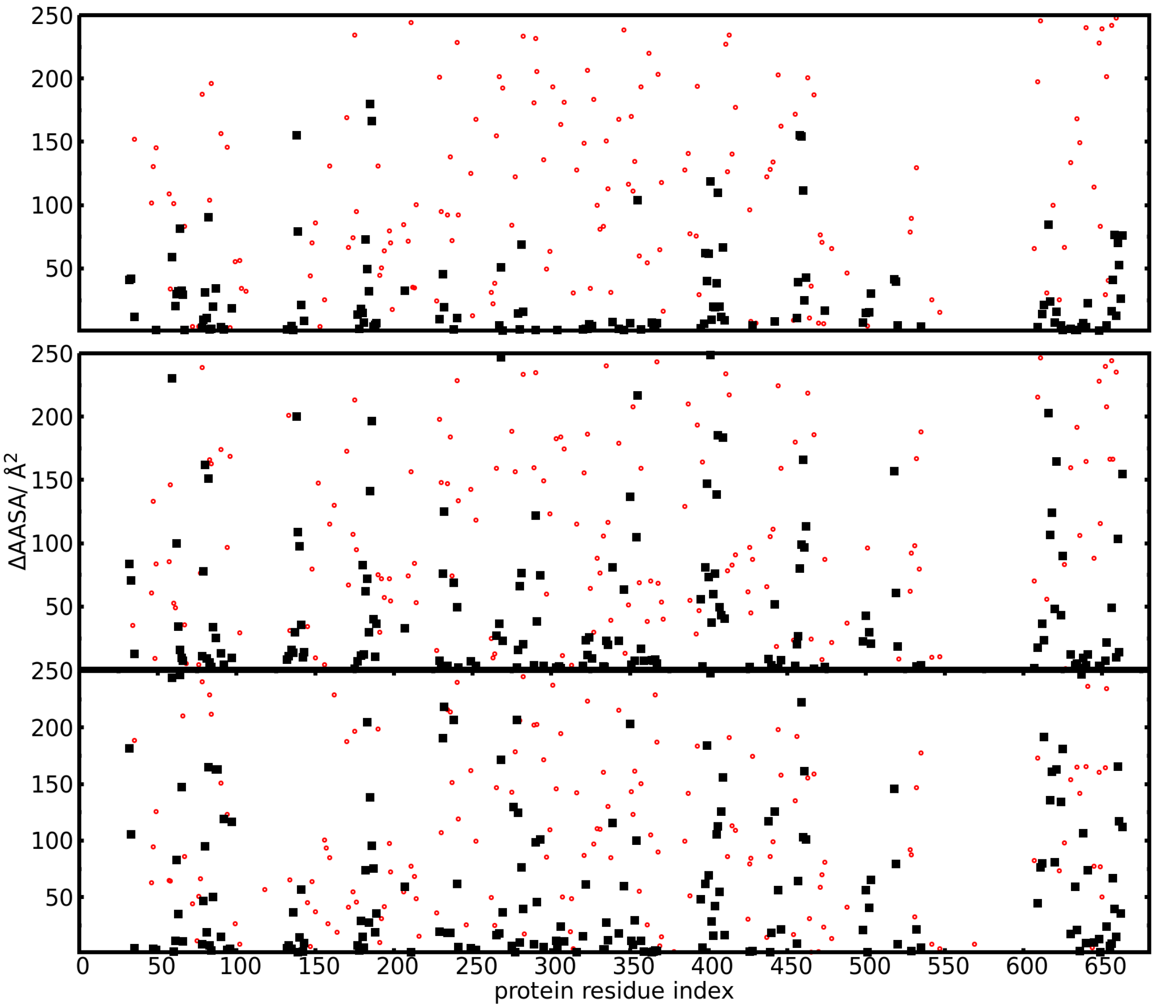

Figure S8. The average (black squares) and maximum (red circles) values of the antibody accessible surface area (AASA) of the protein residues from the HREX simulation for protomers M1 (top), M2 (middle) and M3 (bottom). The probe radius was 10.0 Å and the accuracy is 0.5.

References

1. Pancera M*, et al.* Structure and immune recognition of trimeric pre-fusion HIV-1 Env. *Nature* **514**, 455-461 (2014).

2. Sanders RW*, et al.* A Next-Generation Cleaved, Soluble HIV-1 Env Trimer, BG505 SOSIP.664 gp140, Expresses Multiple Epitopes for Broadly Neutralizing but Not Non-Neutralizing Antibodies. *PLoS Pathog.* **9**, e1003618 (2013).

3. Biasini M*, et al.* SWISS-MODEL: modelling protein tertiary and quaternary structure using evolutionary information. *Nucleic Acids Res.* **42**, W252-W258 (2014).

4. Olsson MHM, Søndergaard CR, Rostkowski M, Jensen JH. PROPKA3: Consistent Treatment of Internal and Surface Residues in Empirical pKa Predictions. *J. Chem. Theory Comput.* **7**, 525-537 (2011).

5. Davis IW*, et al.* MolProbity: all-atom contacts and structure validation for proteins and nucleic acids. *Nucleic Acids Res.* **35**, W375-W383 (2007).

6. Kong L, Wilson IA, Kwong PD. Crystal structure of a fully glycosylated HIV-1 gp120 core reveals a stabilizing role for the glycan at Asn262. *Proteins: Struct., Funct., Bioinf.* **83**, 590-596 (2015).

7. Pancera M*, et al.* Structural basis for diverse N-glycan recognition by HIV-1–neutralizing V1–V2–directed antibody PG16. *Nat. Struct. Mol. Biol.* **20**, 804-813 (2013).

8. Kong L*, et al.* Supersite of immune vulnerability on the glycosylated face of HIV-1 envelope glycoprotein gp120. *Nat. Struct. Mol. Biol.* **20**, 796-803 (2013).

9. Julien J-P*, et al.* Crystal Structure of a Soluble Cleaved HIV-1 Envelope Trimer. *Science* **342**, 1477-1483 (2013).

10. Pejchal R*, et al.* A Potent and Broad Neutralizing Antibody Recognizes and Penetrates the HIV Glycan Shield. *Science* **334**, 1097-1103 (2011).

11. McLellan JS*, et al.* Structure of HIV-1 gp120 V1/V2 domain with broadly neutralizing antibody PG9. *Nature* **480**, 336-343 (2011).

12. Garces F*, et al.* Structural Evolution of Glycan Recognition by a Family of Potent HIV Antibodies. *Cell* **159**, 69-79 (2014).

13. Horiya S, MacPherson IS, Krauss IJ. Recent strategies targeting HIV glycans in vaccine design. *Nat. Chem. Biol.* **10**, 990-999 (2014).

14. Guvench O*, et al.* CHARMM Additive All-Atom Force Field for Carbohydrate Derivatives and Its Utility in Polysaccharide and Carbohydrate–Protein Modeling. *J. Chem. Theory Comput.* **7**, 3162-3180 (2011).

15. Raman EP, Guvench O, MacKerell AD, Jr. CHARMM Additive All-Atom Force Field for Glycosidic Linkages in Carbohydrates Involving Furanoses. *J. Phys. Chem. B* **114**, 12981-12994 (2010).

16. Guvench O, Hatcher E, Venable RM, Pastor RW, MacKerell AD, Jr. CHARMM Additive All-Atom Force Field for Glycosidic Linkages between Hexopyranoses. *J. Chem. Theory Comput.* **5**, 2353-2370 (2009).

17. Guvench O*, et al.* Additive Empirical Force Field for Hexopyranose Monosaccharides. *J. Comput. Chem.* **29**, 2543-2564 (2008).

18. Neria E, Fischer S, Karplus M. Simulation of activation free energies in molecular systems. *J. Chem. Phys.* **105**, 1902-1921 (1996).

19. Reiher WE. *Theoretical studies of hydrogen bonding* (1985).

20. Jorgensen WL, Chandrasekhar J, Madura JD, Impey RW, Klein ML. Comparison of simple potential functions for simulating liquid water. *J. Chem. Phys.* **79**, 926-935 (1983).

21. Yang M, Angles d'Ortoli T, Sawen E, Jana M, Widmalm G, MacKerell AD. Delineating the conformational flexibility of trisaccharides from NMR spectroscopy experiments and computer simulations. *Phys. Chem. Chem. Phys.* **18**, 18776-18794 (2016).

22. Guvench O, MacKerell AD, Jr. Computational Fragment-Based Binding Site Identification by Ligand Competitive Saturation. *PLoS Comput. Biol.* **5**, e1000435 (2009).

23. Raman EP, Yu W, Lakkaraju SK, MacKerell AD. Inclusion of Multiple Fragment Types in the Site Identification by Ligand Competitive Saturation (SILCS) Approach. *J. Chem. Inf. Model.* **53**, 3384-3398 (2013).

24. Stewart-Jones Guillaume BE*, et al.* Trimeric HIV-1-Env Structures Define Glycan Shields from Clades A, B, and G. *Cell* **165**, 813-826 (2016).

25. Kong L*, et al.* Complete epitopes for vaccine design derived from a crystal structure of the broadly neutralizing antibodies PGT128 and 8ANC195 in complex with an HIV-1 Env trimer. *Acta Cryst.* **D71**, 2099-2108 (2015).

26. Kunik V, Peters B, Ofran Y. Structural Consensus among Antibodies Defines the Antigen Binding Site. *PLoS Comput. Biol.* **8**, e1002388 (2012).

27. Kunik V, Ashkenazi S, Ofran Y. Paratome: an online tool for systematic identification of antigen-binding regions in antibodies based on sequence or structure. *Nucleic Acids Res.*, (2012).

28. Lemkul JA, Lakkaraju SK, MacKerell AD. Characterization of Mg2+ Distributions around RNA in Solution. *ACS Omega* **1**, 680-688 (2016).

29. Lee Jeong H, de Val N, Lyumkis D, Ward Andrew B. Model Building and Refinement of a Natively Glycosylated HIV-1 Env Protein by High-Resolution Cryoelectron Microscopy. *Structure* **23**, 1943-1951 (2015).

30. Lee JH, Ozorowski G, Ward AB. Cryo-EM structure of a native, fully glycosylated, cleaved HIV-1 envelope trimer. *Science* **351**, 1043-1048 (2016).

31. Kong R*, et al.* Fusion peptide of HIV-1 as a site of vulnerability to neutralizing antibody. *Science* **352**, 828-833 (2016).

32. Diskin R*, et al.* Restricting HIV-1 pathways for escape using rationally designed anti–HIV-1 antibodies. *J. Exp. Med.* **210**, 1235-1249 (2013).

33. Scharf L*, et al.* Antibody 8ANC195 Reveals a Site of Broad Vulnerability on the HIV-1 Envelope Spike. *Cell Rep.* **7**, 785-795 (2014).

34. Scharf L, Wang H, Gao H, Chen S, McDowall Alasdair W, Bjorkman Pamela J. Broadly Neutralizing Antibody 8ANC195 Recognizes Closed and Open States of HIV-1 Env. *Cell* **162**, 1379-1390 (2015).

35. Jardine JG*, et al.* Minimally Mutated HIV-1 Broadly Neutralizing Antibodies to Guide Reductionist Vaccine Design. *PLoS Pathog.* **12**, e1005815 (2016).

36. Garces F*, et al.* Structural Evolution of Glycan Recognition by a Family of Potent HIV Antibodies. *Cell* **159**, 69-79.

37. Berman HM*, et al.* The Protein Data Bank. *Nucleic Acids Res.* **28**, 235-242 (2000).
